# Evaluating foundation models on official German medical licensing examinations: Implications for high-stakes assessment and AI-assisted medical education

**DOI:** 10.1038/s41746-026-03082-7

**Published:** 2026-08-31

**Authors:** Lasse Cirkel, Johannes Knitza, Volker Schillings, Alexander Oksche, Jan Carl Becker, Sebastian Kuhn

**Affiliations:** 1 Marburg University, Institute for Digital Medicine, Marburg, Germany; 2 Marburg University, Institute of Artificial Intelligence in Medicine, Marburg, Germany; 3 German Institute for State Examinations in Medicine, Pharmacy, Dentistry and Psychotherapy (IMPP), Mainz, Germany; 4 Justus Liebig University Giessen, Rudolf-Buchheim-Institute of Pharmacology, Giessen, Germany

**Keywords:** Computational biology and bioinformatics, Health care, Mathematics and computing, Medical research

## Abstract

We evaluated proprietary and open-weight foundation models on 24 German medical licensing examinations (2019–2024), including 7485 items and response data from 119,878 sittings. For fair comparison, the eight vision-capable models were evaluated on the full benchmark and all thirteen on a shared text-only subset. On the full benchmark, Gemini 3.1 Pro achieved the highest overall accuracy, reaching 99.31% on the first (M1) and 98.37% on the second (M2) examination. On the shared text-only subset, proprietary frontier models performed at near-ceiling levels, several open-weight models (including GLM-5 and DeepSeek V3.2-Thinking) were highly competitive, and even compact ones exceeded mean student performance. Image-present items were more difficult for both students and models, but the associated decline was disproportionately larger for models than for students. Human- and model-defined difficulty subsets showed limited overlap, and model-hard subsets revealed residual differences among top systems. These findings underscore the rapid progress of these models, particularly open-weight systems, and the value of official German medical licensing examinations as a restricted-access benchmark with reduced public exposure. They carry implications for high-stakes assessment and AI-assisted medical education, notably multimodal assessment, human-aligned educational tools, and privacy-preserving local deployment.

## Introduction

Large language models (LLMs) are rapidly advancing from experimental systems to increasingly capable tools for healthcare-related tasks. Early milestones were marked by general-purpose models passing the United States Medical Licensing Examination (USMLE) without domain-specific fine-tuning^1,2^. Since then, performance on medical examinations has improved substantially, with contemporary systems achieving passing or near-ceiling scores across multiple national licensing and specialist examinations^3^. In parallel, these models are increasingly being explored for clinical applications such as documentation support and medical question answering^4,5^. However, rising scores on established benchmarks also create an evaluation problem: once performance approaches saturation, benchmark results become less informative about true medical generalization and less useful for distinguishing among frontier systems.

This problem is not unique to medical AI, but reflects a broader pattern in AI evaluation as leading systems converge toward ceiling performance on widely used benchmarks^6^. The same shift is now evident in medicine, where reasoning-enabled models and recent frontier systems achieve very high scores on several established public benchmarks^5,7–9^. Although such results document substantial capability gains, they also compress performance differences at the top end and make aggregate benchmark accuracy less informative for comparing leading systems or tracking further progress. This has increased the need for more discriminative medical evaluations that remain informative in the near-ceiling regime.

However, increasing benchmark difficulty alone does not resolve another central evaluation problem: public exposure. Modern foundation models are trained on large-scale web-crawled corpora and other broad data sources, meaning that publicly accessible medical test sets may be represented directly or indirectly in training data. For example, Gallifant et al. reported that 99.21% of the MedQA test split was detectable in Dolma 1.6, which they used as an open pretraining corpus proxy, based on 8-gram overlap screening^10^. More broadly, benchmark data contamination is increasingly recognized as a substantial source of evaluation bias, and its reliable detection remains methodologically challenging^11^. Although public-benchmark hardening strategies such as question rephrasing and option augmentation can increase difficulty and may reduce exact overlap^12^, they do not eliminate the fact that such resources remain rooted in publicly accessible source material; moreover, rephrased samples can make contamination harder to detect rather than irrelevant^13^. As a result, strong performance on public medical benchmarks can overestimate true generalization to previously unseen exam-style or clinical questions. Restricted-access evaluation sets are therefore particularly valuable because they reduce the risk of direct benchmark contamination and provide a more stringent test of medical reasoning on items that are less likely to have appeared in pre-training data.

A further limitation of current evidence is its narrow coverage of language, region, and medical training context. Most established medical benchmarks are English-language and aligned with US-centric educational and guideline environments, leaving open how well frontier models generalize to region-specific standards in other languages. The German medical licensing examinations therefore provide a particularly stringent test case because they are administered in German and reflect national curricular priorities and guideline-concordant practice. Earlier studies evaluating LLMs on these examinations or on substantial subsets of their questions^14–19^ provided useful proof-of-concept observations. However, they relied on examination items accessed through secondary educational platforms rather than on the authoritative primary dataset provided directly by the German Institute for State Examinations in Medicine, Pharmacy, Dentistry and Psychotherapy (IMPP), and they were conducted without official cooperation with IMPP. Consequently, they could not benchmark models on the authoritative source material itself or consistently leverage official item-level metadata within a controlled evaluation pipeline. Methodological handling also varied across studies, including heterogeneous treatment of media-containing questions, the absence of an explicit vignette-aware strategy for fair text-only subset construction, interface-based rather than fully standardized programmatic evaluation workflows in some cases, and comparatively narrow model coverage focused mainly on early ChatGPT versions and a small number of other commercial systems.

Current evidence also incompletely captures multimodal medical reasoning. Many medically relevant tasks require the joint interpretation of textual and visual information, including clinical photographs, radiological images, histopathology, electrocardiograms, and scientific figures^20^. This is no longer a peripheral issue confined to specialized imaging systems, because leading general-purpose foundation models are now natively multimodal and are increasingly evaluated for healthcare use. Yet most established medical benchmarks remain predominantly text-based, and although recent multilingual multimodal medical-examination benchmarks have begun to address this gap^21,22^, evidence from official examination settings with corresponding human performance data remains limited. Moreover, multimodal evaluation requires separating text-only from image-present items rather than relying on aggregate examination accuracy alone. As a result, it remains unclear how current models perform on official medical examination items that include visual material alongside text, and whether image-present items reveal limitations that are obscured in predominantly text-based evaluation.

A further translational consideration concerns deployment under real-world governance and data-protection constraints. Although the strongest multimodal performance is currently led by proprietary, API-hosted systems, their use may be restricted in healthcare environments where sensitive medical information cannot readily be processed through external cloud services. This is particularly relevant in Europe, where data-protection, governance, and data-sovereignty requirements are often stringent. Locally deployable open-weight models, therefore, represent an important alternative, yet large-scale comparative evidence on their medical reasoning performance relative to proprietary frontier systems remains limited.

Here, we evaluate proprietary and locally deployable open-weight foundation models on the first (M1, taken after the second year) and second (M2, taken after the fifth year) German medical licensing examinations within the six-year medical curriculum, in direct cooperation with IMPP. Both examinations consist of written single-best-answer multiple-choice items with five response options (A–E) and are administered twice yearly (spring and fall), with approximately 10,000 annual examination sittings in each of M1 and M2. The study is based on a restricted-access dataset of 7485 official examination items from 2019 to 2024, reducing the risk of direct benchmark contamination relative to publicly accessible medical question-answering resources. To enable fair comparison across heterogeneous model classes, we distinguish a shared text-only subset from image-present items. Because a substantial proportion of M2 items is embedded in sequentially linked clinical case vignettes, in which several consecutive items share a common clinical scenario, we apply a conservative vignette-aware exclusion rule to prevent within-vignette leakage in text-only analyses. We further compare proprietary frontier systems with deployment-relevant open-weight alternatives and relate model difficulty to official student difficulty indices at the item level. Finally, to retain discriminatory power in the near-ceiling regime, we define difficulty-enriched text-only subsets based on psychometric and model-derived difficulty. Together, these analyses provide a rigorous evaluation framework for assessing contemporary foundation models on official German medical licensing examinations across language, modality, and deployment context, with broader implications for high-stakes assessment and AI-assisted medical education.

## Results

### Overall performance on the full benchmark in vision-capable models

**Fig. 1:**
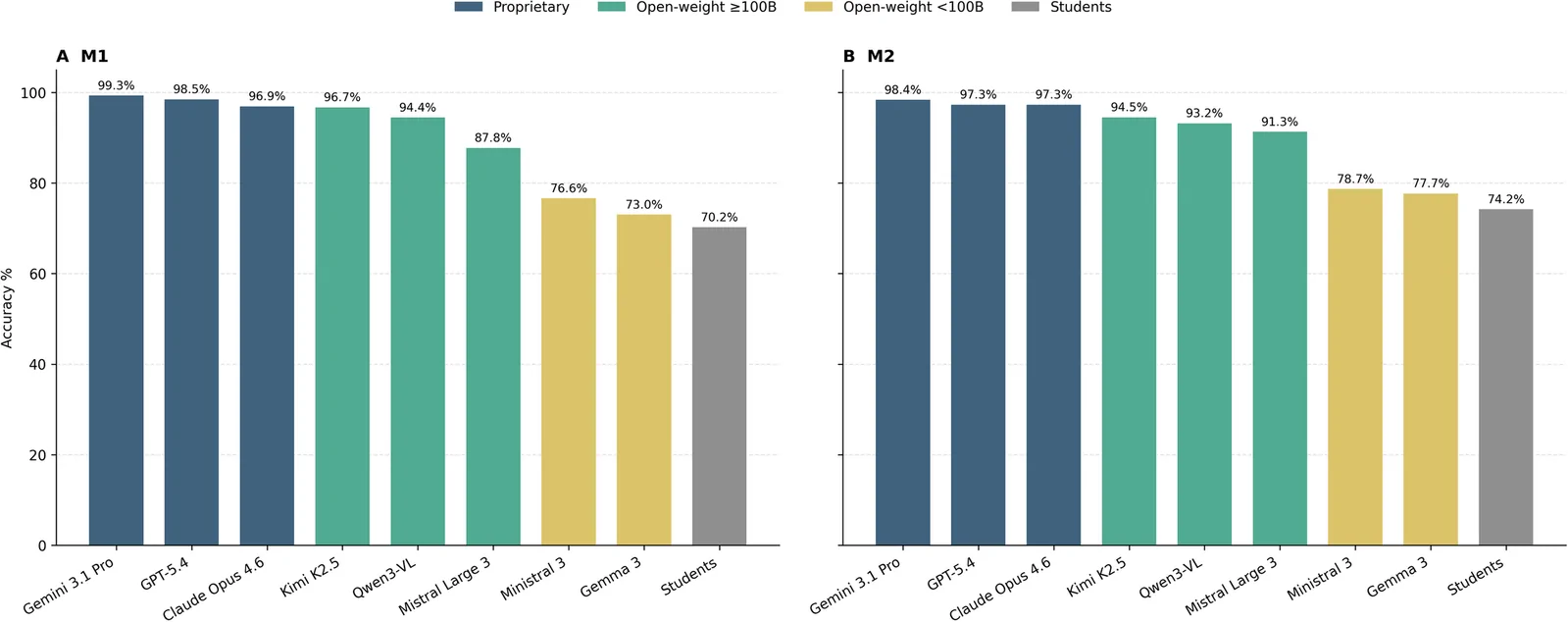
Overall performance on the full benchmark in vision-capable models. Ranked accuracy for vision-capable models on the full benchmark, defined as all retained items, on (**A**) M1 and (**B**) M2, aggregated across examinations and weighted by item counts. The gray bar in each panel indicates mean student accuracy across the corresponding item set. Full numerical results (*k*/*n*, accuracy, and 95% Wilson confidence intervals) are provided in Suppl. Table S2.

To assess end-to-end multimodal performance, we first evaluated vision-capable models on the full benchmark, defined as all retained items for the respective examination set (Fig. 1A,B). On M1 (*n* = 3747), Gemini 3.1 Pro achieved the highest overall accuracy at 99.31% (95% CI 98.99–99.53), followed by GPT-5.4 at 98.48% (95% CI 98.03–98.82) and Claude Opus 4.6 at 96.93% (95% CI 96.33–97.44). Among open-weight vision-capable models, Kimi K2.5 reached 96.72% (95% CI 96.10–97.24), followed by Qwen3-VL at 94.45% (95% CI 93.67–95.14) and Mistral Large 3 at 87.75% (95% CI 86.66–88.76).

On M2 (*n* = 3738), Gemini 3.1 Pro again achieved the highest overall accuracy at 98.37% (95% CI 97.91–98.73). Claude Opus 4.6 and GPT-5.4 followed at 97.30% each (95% CI 96.73–97.77). Among open-weight vision-capable models, Kimi K2.5 achieved 94.52% (95% CI 93.74–95.20), followed by Qwen3-VL at 93.18% (95% CI 92.32–93.94) and Mistral Large 3 at 91.33% (95% CI 90.39–92.19). Smaller vision-capable open-weight models, including Gemma 3 and Ministral 3, showed lower overall performance and are included in Fig. 1; full numerical results for all evaluated models are provided in Suppl. Table S2.

### Performance on the shared text-only subset across all models

**Fig. 2:**
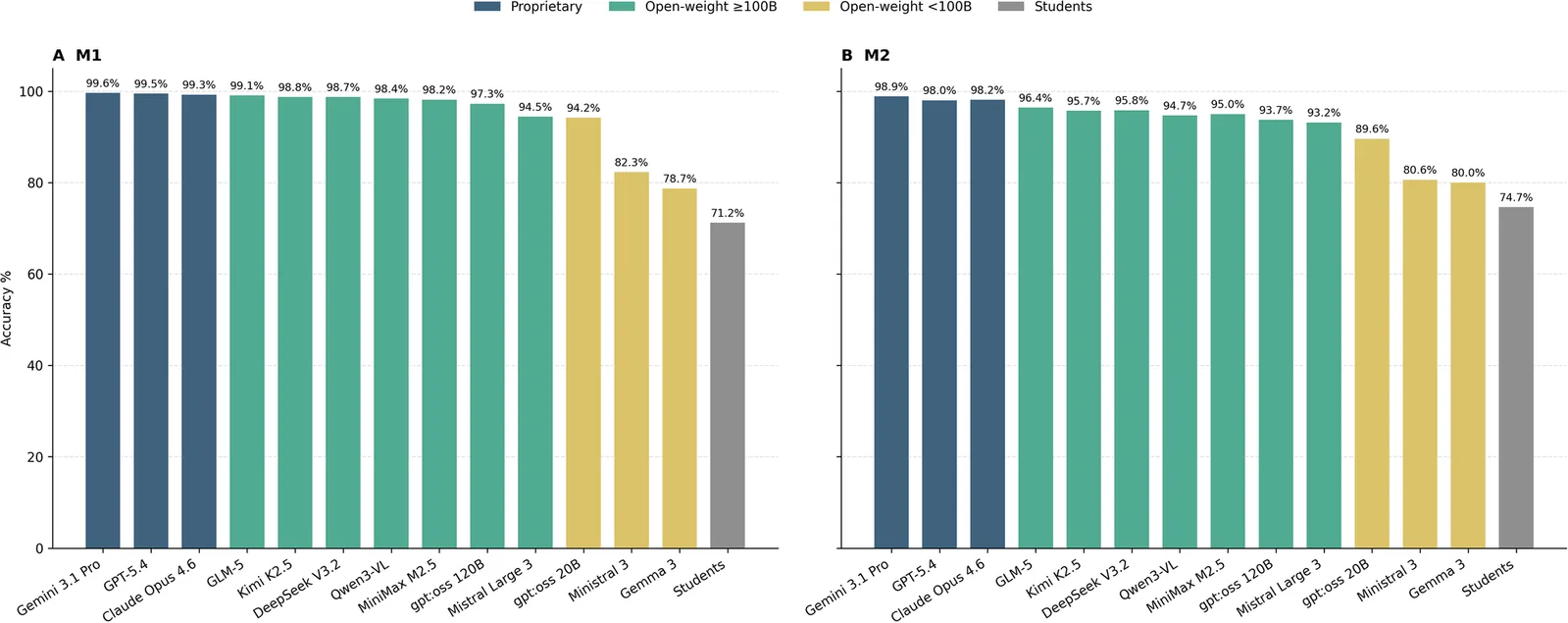
Performance on the shared text-only subset across all models. Ranked text-only accuracy for all evaluated models on (**A**) M1 and (**B**) M2, aggregated across examinations and weighted by item counts. Models are grouped by access type and size tier. The gray bar in each panel indicates mean student accuracy across the corresponding text-only item set. Full numerical results (*k*/*n*, accuracy, and 95% Wilson confidence intervals) are provided in Suppl. Table S2.

Because not all evaluated models supported image input, we next compared all models on the shared text-only subset (M1 *n* = 3227; M2 *n* = 3244), which provides the fairest common basis for cross-model comparison (Fig. 2A, B).

On M1 text-only items, Gemini 3.1 Pro achieved the highest accuracy at 99.63%, followed by GPT-5.4 at 99.50%, Claude Opus 4.6 at 99.26%, GLM-5 at 99.10%, Kimi K2.5 at 98.76%, DeepSeek V3.2-Thinking at 98.73%, and Qwen3-VL at 98.42%. Additional strong performance was observed for MiniMax M2.5 at 98.17% and gpt:oss 120B at 97.27%, whereas Mistral Large 3 and gpt:oss 20B reached 94.45% and 94.24%, respectively.

On M2 text-only items, Gemini 3.1 Pro again ranked first at 98.86%, followed by Claude Opus 4.6 at 98.15% and GPT-5.4 at 98.03%. The strongest open-weight models on this subset were GLM-5 at 96.42%, DeepSeek V3.2-Thinking at 95.84%, Kimi K2.5 at 95.75%, MiniMax M2.5 at 95.01%, and Qwen3-VL at 94.73%. gpt:oss 120B and Mistral Large 3 reached 93.74% and 93.16%, respectively, whereas gpt:oss 20B achieved 89.61% (Fig. 2A, B; Suppl. Table S2).

Several large open-weight models also achieved very high accuracy on the shared text-only subset, particularly in M1.

### Modality-stratified performance and comparison with the human reference

**Fig. 3:**
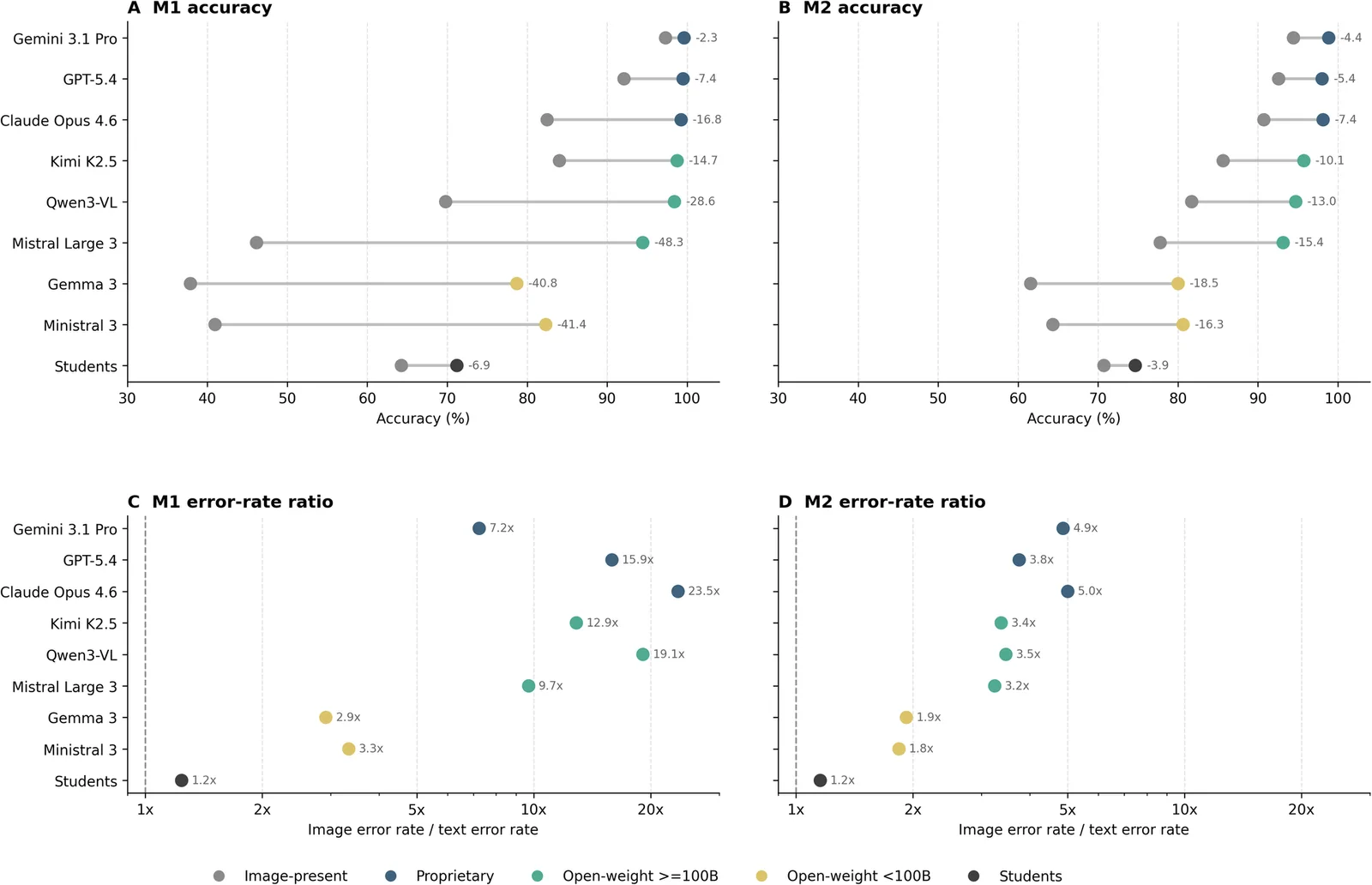
Modality-stratified performance reveals a persistent text–image gap. **A**, **B** Accuracy on the shared text-only subset versus the image-present subset for vision-capable models on (**A**) M1 and (**B**) M2, with the corresponding mean student accuracy shown as a reference. Each model is shown with paired text-only and image-present accuracies connected by a line; numerical labels indicate the absolute text–image gap in percentage points. Models are ordered consistently across panels and grouped by access type and size tier. **C**, **D** Error-rate ratio between image-present and text-only items, defined as (1 − acc_image_)/(1 − acc_text_), for the same models on (**C**) M1 and (**D**) M2. Values are shown on a logarithmic scale; the dashed reference line at 1 × corresponds to equal error rates. Full numerical results for absolute accuracies are reported in Suppl. Tables S3 and S4.

We next quantified performance separately on the shared text-only subset and on image-present items for vision-capable models (Fig. 3A, B). Across the evaluated model inventory, accuracy on image-present items was consistently lower than on text-only items.

On M1, Gemini 3.1 Pro achieved 99.63% on text-only items versus 97.31% on image-present items, corresponding to a text–image gap of 2.32 percentage points. GPT-5.4 achieved 99.50% versus 92.12% (gap 7.39 points), and Claude Opus 4.6 achieved 99.26% versus 82.50% (gap 16.76 points). Among open-weight models, Kimi K2.5 reached 98.76% on text-only items but 84.04% on image-present items (gap 14.72 points), whereas Qwen3-VL showed a larger gap, with 98.42% on text-only items versus 69.81% on image-present items (gap 28.61 points). Mistral Large 3 reached 94.45% on text-only items but only 46.15% on image-present items (gap 48.30 points).

On M2, Gemini 3.1 Pro achieved 98.86% on text-only items and 94.44% on image-present items (gap 4.41 points). GPT-5.4 achieved 98.03% versus 92.59% (gap 5.43 points), and Claude Opus 4.6 achieved 98.15% versus 90.74% (gap 7.41 points). Among open-weight models, Kimi K2.5 reached 95.75% on text-only items and 85.65% on image-present items (gap 10.10 points), Qwen3-VL reached 94.73% versus 81.71% (gap 13.02 points), and Mistral Large 3 reached 93.16% versus 77.78% (gap 15.38 points) (Fig. 3A, B; Suppl. Tables S3 and S4).

To contextualize these model-level gaps against the human reference, we computed the corresponding mean student accuracies on the same item sets from the official item-level student correctness proportions. Mean student accuracy was 71.21% on M1 text-only items versus 64.27% on M1 image-present items (gap 6.94 percentage points), and 74.66% on M2 text-only items versus 70.74% on M2 image-present items (gap 3.92 points). Image-present items were therefore also harder for human candidates than text-only items, although direct comparison of absolute percentage-point gaps is limited by floor and ceiling effects.

We additionally quantified the modality-specific performance drop using the error-rate ratio, i.e. the ratio of the error rate on image-present items to the error rate on text-only items, which is invariant to the baseline accuracy level. For human candidates, the error-rate ratio was 1.24 × on M1 and 1.15 × on M2, indicating only a modest increase in error rate from text-only to image-present items. Across all evaluated vision-capable models, the error-rate ratio was substantially larger: on M1, it ranged from 7.2 × (Gemini 3.1 Pro) to 23.5 × (Claude Opus 4.6) for proprietary frontier models, from 9.7 × to 19.1 × for open-weight models ≥ 100B parameters, and from 2.9 × to 3.3 × for open-weight models < 100B parameters. On M2, error-rate ratios ranged from 3.8 × to 5.0 × for proprietary frontier models, from 3.2 × to 3.5 × for open-weight models ≥ 100B parameters, and from 1.8 × to 1.9 × for open-weight models < 100B parameters (Fig. 3C, D).

Taken together, these results show persistent text–image performance gaps across models. Image-present items were also more difficult for human candidates, but the modality penalty was disproportionately larger for models: in absolute terms, all evaluated models except Gemini 3.1 Pro on M1 showed a larger drop than students, and in relative terms the multiplicative increase in error rate was far larger for models (≈ 1.2 × for students versus several-fold for most models). Because this ratio normalizes each solver against its own text-only performance, this contrast indicates that image-present items pose an additional, model-specific difficulty beyond the general difficulty they also present to human candidates. The error-rate ratio is nonetheless sensitive to baseline accuracy and should not be used to rank individual models against one another.

### Item-level correlation between human and model difficulty

We next assessed whether item-level model difficulty aligned with official student difficulty on eligible items from the shared text-only subset. For M1, item-level human difficulty and model difficulty showed a positive correlation (*ρ* = 0.318, 95% CI 0.286–0.346; *n* = 3227 items). For M2, the association was slightly stronger (*ρ* = 0.333, 95% CI 0.302–0.364; *n* = 3244 items). These findings indicate moderate, but incomplete, alignment between human and model difficulty at the item level (Suppl. Fig. S1).

### Difficulty-enriched text-only subsets reveal residual model differences and human–model discordance

**Fig. 4:**
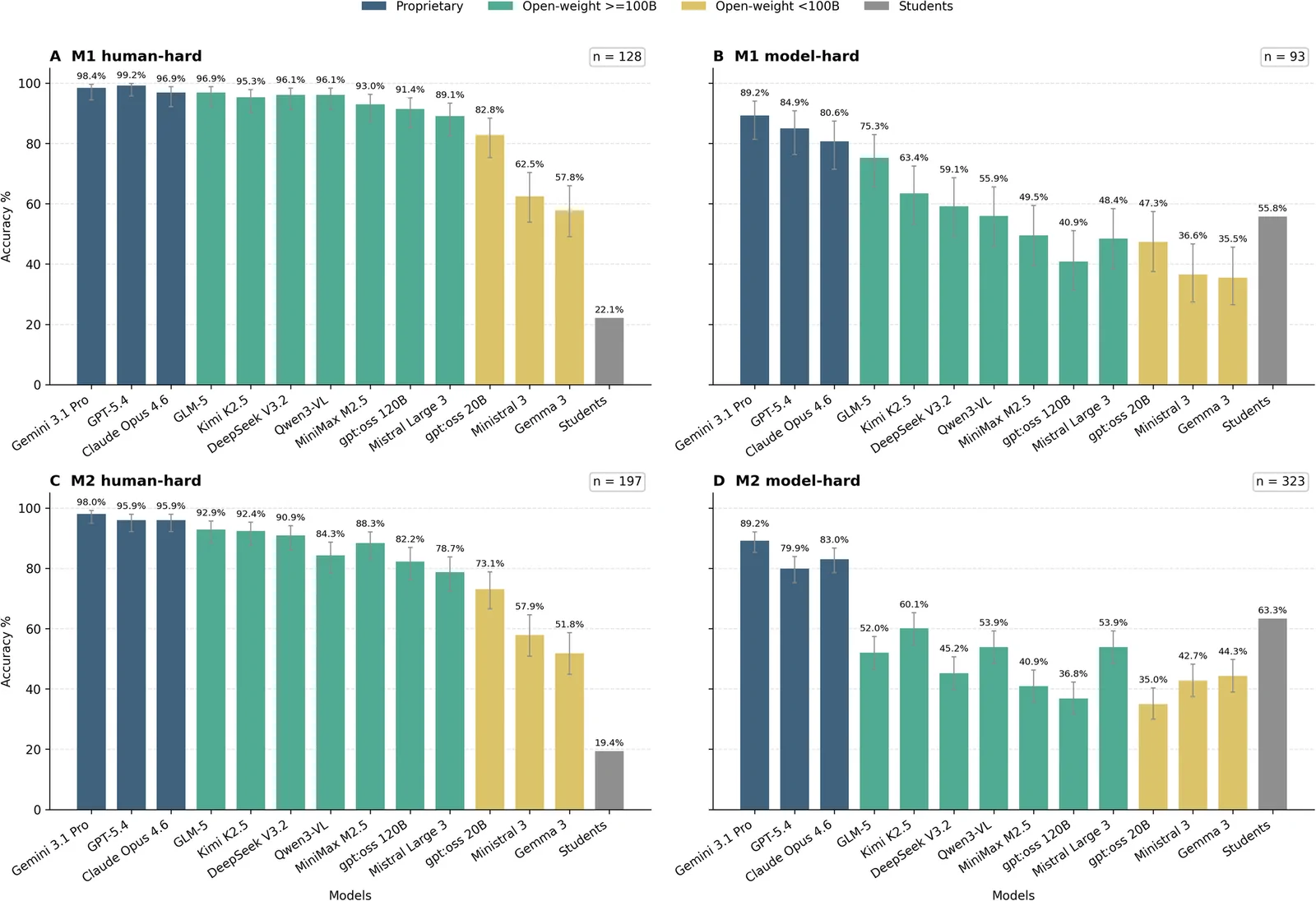
Difficulty-enriched text-only subsets reveal residual performance differences beyond aggregate benchmark accuracy. Performance of evaluated models on difficulty-enriched text-only subsets in M1 and M2. (**A**) M1 human-hard-text, defined as items with student *p* ≤ 0.30; (**B**) M1 model-hard-text, defined as items on which at least two models from the top-tier comparison pool answered incorrectly; (**C**) M2 human-hard-text; and (**D**) M2 model-hard-text. Bars indicate model accuracy, error bars denote 95% Wilson confidence intervals, and the gray bar in each panel shows mean student accuracy for the corresponding subset. The model-hard-text subsets showed stronger descriptive separation among top-performing systems than the human-hard-text subsets. Full numerical results are reported in Suppl. Tables S5 and S6.

Given only moderate alignment between human and model difficulty, we next evaluated difficulty-enriched subsets of the shared text-only benchmark separately for M1 and M2 (Fig. 4). Human-hard-text was defined by an official student *p*-value of ≤ 0.30, whereas model-hard-text comprised items missed by at least two models from the top-tier comparison pool.

In M1, the human-hard-text subset comprised 128 items, with a mean student *p*-value of 0.221. On this subset, GPT-5.4 achieved 99.22%, Gemini 3.1 Pro 98.44%, and Claude Opus 4.6 and GLM-5 each 96.88%. In M2, the human-hard-text subset comprised 197 items, with a mean student *p*-value of 0.194. On this subset, Gemini 3.1 Pro achieved 97.97%, GPT-5.4 and Claude Opus 4.6 each 95.94%, and GLM-5 92.89%.

By contrast, performance was more dispersed across models on the model-hard-text subset. In M1, this subset comprised 93 items, with a mean student *p*-value of 0.558. Gemini 3.1 Pro achieved 89.25%, GPT-5.4 84.95%, Claude Opus 4.6 80.65%, and GLM-5 75.27%. In M2, the model-hard-text subset comprised 323 items, with a mean student *p*-value of 0.633. Gemini 3.1 Pro reached 89.16%, followed by Claude Opus 4.6 at 82.97% and GPT-5.4 at 79.88%; among open-weight models, Kimi K2.5 achieved 60.06%, followed by Mistral Large 3 and Qwen3-VL at 53.87%, and GLM-5 at 52.01% (Fig. 4; Suppl. Tables S5 and S6).

Overlap between the two subset definitions was limited, with 12 shared items in M1 and 40 in M2 (Suppl. Table S7). Cross-classification further showed that most model-hard-text items were not human-hard-text (81/93 in M1 and 283/323 in M2), whereas most human-hard-text items were not model-hard-text (116/128 in M1 and 157/197 in M2).

To characterize what distinguishes these two off-diagonal cells, we asked five of the evaluated language models to describe, in qualitative terms and without knowledge of the selection criteria, the recurring properties and the competence probed by a sample of items from each cell (Set A, items that were model-hard-text but not human-hard-text; Set B, items that were human-hard-text but not model-hard-text; Suppl. Note 1). Across these five models, the two cells were distinguished primarily by the cognitive task type they probe. In M1, the models converged on contrasting the classification of described scenarios into concepts and terminology, with a marked psychosocial component (Set A), against the explanation of mechanisms and the application of scientific principles in the basic natural sciences (Set B); here, the task-type contrast was additionally aligned with a behavioral- versus natural-science disciplinary tilt. In M2, an equally consistent task-type axis emerged that cut across clinical specialties rather than tracking a single subject area: the models broadly converged on characterizing Set A as probing situation-appropriate, procedural, and norm-governed clinical decision-making, namely selecting the appropriate next action within realistic, management-oriented scenarios, with several models additionally noting a recurring legal, administrative, or ethical component. By contrast, Set B was characterized as probing recognition of specific clinical or laboratory patterns and recall of narrowly defined entities, tests, or standard therapies. Because item content is protected, these characterizations are reported as a convergent qualitative impression across models rather than as a quantified agreement metric (Suppl. Note 1).

### Exam-level robustness and empirical probes for direct contamination

To assess robustness across individual examination administrations and to empirically probe for direct benchmark contamination, we examined exam-wise accuracy across the 12 M1 and 12 M2 examinations on the shared text-only subset. Reported pre-training cutoffs of the evaluated models span June 2024 (gpt:oss 20B and gpt:oss 120B) to August 2025 (GPT-5.4), allowing the same exam-wise data to support both a robustness assessment and a temporally structured contamination probe.

Across administrations, the strongest models showed consistently high performance with relatively narrow exam-wise ranges, whereas some lower-performing systems exhibited broader dispersion. On M1, exam-wise text-only accuracy ranged from 98.92% to 100.00% for Gemini 3.1 Pro, from 98.90% to 100.00% for GPT-5.4, and from 98.06% to 100.00% for Claude Opus 4.6. Strong open-weight models also remained comparatively stable, including Kimi K2.5 (97.70% to 99.61%), Qwen3-VL (96.93% to 99.29%), GLM-5 (98.47% to 100.00%), and DeepSeek V3.2-Thinking (97.32% to 99.64%). On M2, Gemini 3.1 Pro ranged from 98.10% to 100.00%, GPT-5.4 from 96.54% to 100.00%, and Claude Opus 4.6 from 96.93% to 100.00%. Among open-weight models, Kimi K2.5 ranged from 93.10% to 98.55%, Qwen3-VL from 93.54% to 96.74%, GLM-5 from 94.64% to 98.85%, and DeepSeek V3.2-Thinking from 93.92% to 99.64%. Full exam-wise heatmaps for all models are provided in Suppl. Fig. S2 (M1) and Suppl. Fig. S3 (M2).

To probe for direct contamination effects, we first examined whether models showed an accuracy drop across the pre-training cutoff boundary. Three evaluated open-weight models have reported cutoffs that fall between the Spring 2024 (administered March–April 2024) and Fall 2024 (administered October 2024) examinations: gpt:oss 20B and gpt:oss 120B (both June 2024) and Gemma 3 (August 2024). If direct memorization of pre-training-resident items contributed substantially to performance, these models should perform measurably worse on Fall 2024 than on Spring 2024 items. The observed Spring-to-Fall 2024 differences on the shared text-only subset were − 0.22 percentage points (pp) (95% CI − 4.32, + 3.79) on M1 and − 1.03 pp (95% CI − 6.14, + 4.04) on M2 for gpt:oss 20B; − 0.14 pp (95% CI − 3.57, + 3.21) on M1 and − 3.35 pp (95% CI − 7.24, + 0.14) on M2 for gpt:oss 120B; and − 0.86 pp (95% CI − 7.84, + 6.08) on M1 and − 2.80 pp (95% CI − 9.66, + 4.01) on M2 for Gemma 3. All six confidence intervals comfortably include zero. Differences of comparable magnitude were observed for proprietary frontier models whose cutoffs fall well after both 2024 administrations (e.g., GPT-5.4 M2 − 3.76 pp; Claude Opus 4.6 M2 − 2.62 pp; Gemini 3.1 Pro M2 − 2.26 pp), indicating that the modest Spring-to-Fall drops observed on M2 reflect an examination-specific difficulty difference affecting all models uniformly rather than a contamination-driven effect on cutoff-critical models. On M1, Spring-to-Fall differences were centered near zero across the inventory. This boundary-crossing test is necessarily restricted to the three open-weight models whose cutoff lies within our examination range; for the remaining models, direct boundary testing is not feasible.

Second, we examined whether examinations from earlier years showed systematically higher accuracy than recent examinations, as would be expected if older items had propagated through web-indexed materials over time. Per-model Spearman correlations between chronological examination index (1–12) and exam-wise accuracy on the shared text-only subset were small in magnitude and not consistently signed across the model inventory (Suppl. Table S8). For M1, correlations ranged from − 0.586 (GPT-5.4) to + 0.357 (gpt:oss 20B) with no systematic pattern by model class or reported cutoff. For M2, correlations ranged from − 0.336 (Mistral Large 3) to + 0.322 (gpt:oss 120B), again with mixed signs across models with both early and late cutoffs. This second probe is necessarily a coarser signal than the boundary-crossing test, but applies to the full model inventory and tests an alternative contamination mechanism (gradual web propagation of older material) rather than direct ingestion of specific item text.

Taken together, exam-wise performance was robust across the 2019–2024 administrations, and the two contamination probes are consistent with low direct benchmark contamination across the evaluated model inventory. Neither approach rules out indirect exposure through paraphrased or restructured material in secondary educational platforms, and reliable corpus-level contamination detection would require access to provider pre-training data, which is not publicly disclosed for most evaluated models.

### Secondary analyses

Among the three evaluated base models without native thinking support, two-stage chain-of-thought prompting improved performance relative to direct constrained answering in both M1 and M2, with the largest gains observed in Ministral 3 (Suppl. Fig. S4; Suppl. Tables S9 and S10). In a separate within-family sub-analysis on a fixed recent text-only subset, performance across successive OpenAI model generations increased from 66.16% for GPT-3.5 Turbo to 98.30% for GPT-5.4, illustrating the rapid compression of benchmark performance toward ceiling levels (Suppl. Fig. S5).

## Discussion

In this study, we benchmarked frontier proprietary and open-weight large language models on official German medical licensing examination items provided through direct cooperation with IMPP. Four main findings emerge. First, performance on the shared text-only subset approached ceiling levels in the strongest models, indicating that aggregate exam accuracy is becoming increasingly compressed as a discriminator of frontier capability. Second, this near-ceiling behavior did not extend to image-present items, where the relative increase in error rate was several-fold larger for models than for human candidates. Third, model difficulty aligned with human difficulty only partially: although item-level human and model difficulty were positively correlated, the association was only moderate, and human-hard-text and model-hard-text subsets showed limited overlap. Fourth, several large open-weight models were already highly competitive on text-only items, and even compact local models exceeded average student performance, underscoring the practical relevance of privacy-preserving deployment candidates.

A central implication of these results is that medical LLM benchmarking is entering a regime in which aggregate text-only accuracy alone is no longer sufficient. On this restricted-access benchmark, several top models now operate at or near the upper end of the human performance range, while our within-family temporal analysis illustrates how quickly this compression can arise over only a few model generations. This broader pattern is increasingly visible across established medical benchmarks, where recent reasoning-enabled systems have already reached near-saturation performance on several widely used evaluations^9^. Together, these findings suggest that once benchmark formats become highly familiar and increasingly solvable by frontier architectures, average performance metrics lose much of their ability to distinguish among top-tier systems. This is particularly relevant beyond English-language settings, because recent multilingual and cross-national analyses suggest that medical LLM performance can vary substantially across languages and examination contexts, with lower accuracy often observed outside English benchmarks^3,23^. These observations further underline the value of region-specific benchmark resources beyond English-language exam settings.

The difficulty-enriched subset analyses further illustrate this limitation of aggregate accuracy. The human-hard-text subset captured items on which student performance approached guessing level, yet the strongest LLMs still answered these questions with very high accuracy. By contrast, the model-hard-text subset showed greater descriptive separation among frontier models; because this subset was defined from the errors of the top-tier model pool, however, this separation is partly circular by construction and should be read as descriptive rather than as an unbiased ranking of the same models. Student performance on these items was only moderately reduced relative to the overall benchmark. These two subset types, therefore, capture different notions of difficulty, and their limited overlap reinforces the conclusion from the correlation analysis: models do not simply reproduce the human hierarchy of difficult items.

The exploratory LLM-assisted characterization of sampled off-diagonal items provides one possible qualitative explanation for this mismatch. Because this characterization was generated by judge models under neutral set labels, rather than by formal author-coded content analysis, it should be interpreted cautiously. Still, the responses consistently pointed to differences in the type of task elicited. In M1, this task-type difference partly overlapped with domain differences between more classificatory or terminology-oriented items and more mechanistic basic-science items. In M2, where both off-diagonal item sets spanned clinically heterogeneous subjects, the contrast was more clearly one of competence type: context-sensitive procedural and norm-governed clinical judgment on one side, and disease-specific recognition or retrieval of established biomedical knowledge on the other. A further contributing factor to the models’ relatively weaker performance on these procedural, norm-governed items (several of which carry a legal, administrative, or ethical component) may be our deliberately neutral primary prompt, which did not state that the items originated from the German medical licensing examinations. Although the German language and item content provided contextual cues, this neutral framing may have left models without the specific jurisdictional context required for guideline- or regulation-dependent decisions, and could be examined in future work by explicitly specifying the examination context in the prompt. This task-type characterization supports the interpretation that model difficulty and student difficulty are related but not interchangeable signals. For medical educators developing AI-assisted tutoring systems, this means that model-derived difficulty estimates should be calibrated against human learner performance rather than treated as direct proxies for student difficulty.

The most clinically relevant weakness revealed by this benchmark was the persistent gap between text-only and image-present performance. Although the strongest proprietary multimodal models remained highly accurate overall, all vision-capable systems performed worse on image-present items than on text-only items, and the performance loss was especially pronounced in several open-weight models. To assess whether this reflected a model-specific limitation or a general property of image-present items, we additionally compared the model-level error rates with the corresponding student-level reference. Image-present items were more challenging for human candidates than text-only items, but this penalty was more pronounced for models. In absolute terms, every evaluated vision-capable model except Gemini 3.1 Pro on M1 showed a greater drop in accuracy on image-present items than human candidates did, despite the models’ near-ceiling performance on text-only items. In relative terms, the difference was even more marked, with the increase in error rate being approximately 1.2-fold for human candidates versus several-fold for most models. Because this ratio normalizes each solver against its own text-only baseline, it suggests that image-present items are not only more challenging in general but also present an additional difficulty specific to the models. However, the ratio is sensitive to baseline accuracy and should not be used to rank individual models’ vision capability. The strongest models (e.g., Gemini 3.1 Pro) remained highly accurate on image-present items, whereas several open-weight vision models declined substantially. This matters because clinically relevant reasoning often requires integrating textual context with visual and other non-text modalities rather than relying on text alone^24,25^. Our findings, therefore, suggest that text-only medical benchmark saturation should not be mistaken for broadly solved medical reasoning. As medical curricula increasingly emphasize integrative, multimodal clinical reasoning, the use of LLMs in educational settings should account for these persistent visual deficits to avoid giving learners a misleading impression of model competence across modalities. This interpretation is consistent with prior work showing that high multimodal multiple-choice accuracy can still conceal substantial weaknesses in image-grounded reasoning and rationale quality^26^. A model may perform near ceiling on text-based exam questions while still showing meaningful deficits when visual interpretation becomes necessary for the correct answer. One plausible hypothesis for the generally smaller text–image gap observed in most models in M2 than in M1 is that M2 image-present items may remain partially answerable from the accompanying clinical vignette and textual context, whereas image-present items in M1 may more often depend directly on the visual content itself, for example, in questions involving structural, schematic, or diagrammatic interpretation. Because we did not perform formal image-type or image-necessity annotation, this explanation remains a hypothesis rather than an established mechanism; testing it would require such annotation, which is beyond the scope of the present study.

These findings also have translational implications. For medical education and assessment, near-ceiling performance on text-only licensing-style items suggests that conventional multiple-choice formats may become less informative as stand-alone indicators of competence in the AI era, whereas multimodal and difficulty-enriched items may retain greater discriminatory value. Accordingly, the educational challenge is not simply whether models can pass exams, but how assessment formats, AI literacy, and governance should evolve in response. Proprietary frontier systems retained the strongest overall and multimodal performance, but several large open-weight models came close to these systems on shared text-only tasks, and some compact local models already exceeded average student performance. This is particularly relevant for healthcare environments and medical universities in which external API use is constrained by privacy, governance, or data-sovereignty requirements regarding patient and student data. Under such conditions, the practical question is not only which model ranks first overall, but which classes of models are already strong enough to support local experimentation, educational use, or privacy-sensitive clinical prototyping. The strong performance of compact models further supports the feasibility of privacy-preserving, locally deployed AI tutoring systems within university infrastructures. In educational settings, these deployment choices are also governance choices. Institutions must balance capability with privacy, data sovereignty, and academic integrity, particularly when students use AI systems for preparation on clinically framed materials. The growing strength of locally deployable open-weight models makes this trade-off increasingly actionable for universities and examination-adjacent training environments.

At the same time, benchmark performance should not be conflated with translational readiness: recent commentary has emphasized that medical LLM evaluations can create an overly optimistic picture when conclusions are drawn too directly from benchmark scores alone^27^. Conversely, more workflow-near studies are beginning to use more realistic endpoints, including prospective randomized evaluations of physician performance on clinically framed patient-care tasks and clinician–AI collaborative diagnostic workflows^28,29^. In this context, our benchmark is best understood as a capability signal and comparative screening instrument rather than a substitute for workflow-level clinical validation. The multimodal results further suggest that the remaining gap between open-weight and proprietary systems is currently more consequential in image-dependent settings than in text-only ones.

An additional strength of this study is that these results were obtained under a deliberately constrained evaluation design. Performance reflects relatively raw model capability under standardized conditions: zero-shot prompting, single-pass inference, no retrieval augmentation, no external tools, no interactive decomposition, and no majority-vote or ensemble strategy. This makes the benchmark conservative in one sense, because some models might achieve even higher scores in optimized agentic pipelines, but it also isolates intrinsic model capability more cleanly than heavily scaffolded evaluation setups. In this respect, the study does not primarily show what can be achieved with complex engineering around a model; rather, it shows how strong current systems already are on a restricted-access medical benchmark without such support.

A further strength is that the benchmark was constructed directly from the authoritative primary examination dataset in cooperation with IMPP. The underlying examination items arise from an extensive expert-driven development and quality-assurance process involving appointed subject-matter experts, iterative revision, formal review before exam deployment, and post-exam review of items challenged by students or flagged by statistical irregularities^30,31^. This strengthens the benchmark’s value as an evaluation resource derived from a highly curated national examination process.

Several limitations should be considered. First, performance on a licensing-style multiple-choice examination is not equivalent to safe or effective clinical deployment. Our benchmark does not evaluate open-ended clinical generation, longitudinal reasoning over evolving patient data, calibration, uncertainty communication, or downstream effects on decision quality and patient outcomes. Similarly, the multimodal analysis remains exam-based rather than workflow-based and should not be interpreted as a comprehensive assessment of real-world clinical imaging use. Second, although the restricted-access nature of the dataset reduces the risk of direct benchmark contamination relative to public web-exposed resources, indirect exposure through licensed reproductions or secondary educational materials cannot be fully excluded. Two empirical probes based on the temporal structure of the examination series and the reported pre-training cutoffs of evaluated models did not reveal evidence of a substantial direct contamination effect across the evaluated model inventory, but cannot rule out indirect exposure through paraphrased or restructured secondary materials; reliable corpus-level contamination detection would require access to pre-training data, which is not publicly disclosed for most evaluated models. Third, the benchmark is intentionally national and language-specific. This is a major strength for assessing regional generalization beyond English, US-centric benchmarks, but it also means that the findings should not be assumed to transfer unchanged to other healthcare systems or examination frameworks. Fourth, the difficulty-enriched subsets were operationally defined to recover discrimination under near-ceiling conditions, and alternative subset definitions could also be justified. The model-hard-text subsets should therefore be interpreted as exploratory and descriptive difficulty-enriched subsets rather than externally validated benchmark strata. In addition, model rankings should be interpreted descriptively; exhaustive paired model-versus-model superiority testing was not performed, and each model–item pair was evaluated once without repeated sampling or majority voting. Small accuracy differences among frontier systems in the near-ceiling regime may therefore be sensitive to stochastic response variability and should not be overinterpreted. Fifth, we did not perform a formal subject-level, image-type, image-necessity, or human-coded qualitative item-content analysis. The exploratory LLM-assisted characterization of human–model discrepancy items provides qualitative context for the observed mismatch, but it should not be interpreted as a validated qualitative coding study. It was based on sampled text-only items, used judge models from the evaluated model pool, did not include human rater coding or inter-rater agreement, and the protected item texts cannot be reproduced for external item-level auditing. Accordingly, the fine-grained item-level sources of the observed mismatch between human and model difficulty, as well as modality-specific subgroup effects among image-present items, remain to be characterized in future work. Finally, the vignette-aware exclusion rule prevented within-vignette visual leakage but also restricted the prompt context provided to models: for items embedded in case vignettes, models received the vignette text and preceding items but not subsequent items from the same vignette. Human candidates, by contrast, see the entire vignette including all linked items, and may use information from later items as cues for earlier ones, which may slightly underestimate model performance relative to the human reference for these items.

Overall, official German medical licensing examinations provide a stringent, restricted-access benchmark, with reduced direct public exposure and explicit contamination-probe analyses, for evaluating medical foundation models in a non-English, deployment-relevant setting. On this benchmark, frontier systems approached near-ceiling performance on the shared text-only subset, whereas performance on image-present items remained consistently lower, indicating that multimodal medical reasoning is still substantially less mature than text-based exam performance. These findings suggest that aggregate benchmark accuracy alone is no longer sufficient for frontier model evaluation. Future medical AI benchmarks should therefore be modality-stratified, difficulty-enriched, and explicitly designed for fair comparison across heterogeneous model classes, while translational assessment should increasingly consider the growing relevance of strong open-weight models for privacy-sensitive local deployment.

## Methods

### Dataset and examination structure

We benchmarked LLM performance on official German medical licensing examination items provided by IMPP, covering all spring and fall examination sessions from 2019 through 2024. The dataset comprised the first (M1) and second (M2) of the three German medical state examinations, yielding 12 M1 and 12 M2 examinations across the six-year study period. Each examination contains 320 multiple-choice items, corresponding to 7680 nominal item positions before post-examination exclusions.

Following each examination, candidates may formally challenge items perceived as ambiguous, flawed or factually incorrect. Challenged items are reviewed by the responsible expert committees, and a small number of items may subsequently be invalidated and removed from official scoring. All such invalidated items were excluded before benchmarking and scoring, such that model performance was evaluated only against items retained in the official IMPP answer key. Across the included examinations, the number of officially invalidated items varied by exam; exact per-examination counts are reported in Suppl. Table S1.

After removal of officially invalidated items, the final benchmark comprised 7485 evaluated items, including 3747 M1 items and 3738 M2 items. Throughout this study, text-only denotes the shared analysis subset used for fair cross-model benchmarking, whereas image-present denotes items that themselves included at least one image. In M1, all retained items without an image were included in the shared text-only subset, yielding 3227 text-only and 520 image-present items. In M2, text-only classification was stricter because many items are embedded in sequentially linked case vignettes. Once an image occurred in any item within a vignette, that item and all subsequent items from the same vignette were excluded from the shared text-only subset, even if those later items did not contain an image themselves. This conservative rule was applied to avoid within-vignette leakage from earlier visual information. Accordingly, the shared M2 text-only subset comprised 3244 items, while 432 items were image-present. A small additional group of 62 M2 items had no image of their own but was excluded from text-only analyses under this vignette-aware rule; these items remained part of full-benchmark analyses in vision-capable models. A per-examination accounting of retained and excluded items is provided in Suppl. Table S1.

M1 predominantly assesses foundational preclinical biomedical sciences, whereas M2 predominantly assesses clinically applied knowledge across medical specialties. In M2, approximately 55–65% of items occur in sequentially linked case vignettes, whereas approximately 35–45% are single questions linked to an individual case context. All examination items followed a single-best-answer multiple-choice format with five response options (A–E) and exactly one correct answer according to the official IMPP answer key. All content was in German and reflected German medical education standards and guideline-concordant practice.

Items may include one or more figures, such as clinical photographs, radiological images, histopathology, electrocardiograms or scientific diagrams. For the present study, an item was classified as image-present if the question as evaluated included at least one image.

### Data access and benchmark leakage considerations

This study was conducted in formal cooperation between the Institute for Digital Medicine at Philipps University Marburg and IMPP, the rights-holding examination authority. Beyond access to the examination items themselves, this cooperation provided three advantages that were not available to earlier studies. First, we obtained the official item-level student difficulty indices (*p*-values), which are not available through the secondary providers and are required for the item-level human–model difficulty alignment reported here. Second, we obtained the complete official item set together with the official post-examination invalidation decisions, so that each item could be scored strictly against the official answer key; we evaluated all items and then excluded the officially invalidated ones from scoring (Suppl. Table S1). Third, direct provision enabled standardized, fully automated preprocessing across the entire 2019–2024 item set. By contrast, earlier studies without official IMPP cooperation obtained items indirectly, extracting them individually from secondary platforms, which is more error-prone and typically limited to smaller, manually assembled subsets.

The evaluated dataset is not publicly accessible in its benchmarked form. In the German medical education context, the principal secondary distribution channel for IMPP items is commercial subscription-based exam-preparation services that reproduce official examination items under licensing arrangements with IMPP, and access to these services requires a paid individual account. Because such items are not openly published on the web, the dataset is less exposed to direct inclusion in web-crawled pre-training corpora than fully public medical question-answering benchmarks, which reduces the risk of direct benchmark contamination and provides a more stringent, reduced-exposure evaluation setting. Partial overlap through user-generated study materials cannot be fully excluded. To empirically assess the contribution of direct contamination effects, we additionally performed two complementary post-hoc analyses based on the temporal structure of the examination series and the reported pre-training data cutoffs of the evaluated models.

### Ethics

This study used official examination materials and aggregated, fully anonymized examination statistics provided by IMPP. No identifiable personal data were accessed, and no participants were recruited or contacted. According to institutional requirements, this work did not require ethics committee approval or informed consent.

### Model selection

We evaluated a broad set of LLMs spanning both proprietary, API-hosted systems and locally deployed open-weight models. The model inventory was designed to cover the major frontier systems available at the time of evaluation, together with widely used open-weight architectures across a broad range of parameter scales. This allowed comparison between proprietary reference models and locally deployable alternatives under a shared benchmarking framework. The full model inventory, grouped by access modality and size tier and annotated for vision capability and native thinking support, is provided in Table 1.

**Table 1:** Model inventory and capability annotation. Final evaluated model configurations included in this study

Model family	Evaluated configuration	Vision	Thinking	Cutoff
Open-weight models ( < 100B parameters)				
Gemma 3	27B	Y	N	Aug 2024
Ministral 3	14B	Y	N	n.d.
gpt:oss	20B	N	Y	Jun 2024
Open-weight models (≥ 100B parameters)				
gpt:oss	120B	N	Y	Jun 2024
Qwen3-VL	235B	Y	Y	n.d.
MiniMax M2.5	229B	N	Y	n.d.
Mistral Large 3	675B	Y	N	n.d.
DeepSeek V3.2-Thinking	685B	N	Y	n.d.
GLM-5	744B	N	Y	n.d.
Kimi K2.5	1000B	Y	Y	n.d.
Proprietary models (API-hosted)				
GPT-5.4	gpt-5.4-2026-03-05	Y	Y	Aug 2025
Gemini 3.1 Pro	gemini-3.1-pro-preview	Y	Y	Jan 2025
Claude Opus 4.6	claude-opus-4-6	Y	Y	May 2025

Vision indicates native multimodal image input. Thinking indicates an explicit native reasoning mode, enabled at the highest available setting when supported. Cutoff indicates the approximate pre-training data cutoff as reported by the respective model provider in their official documentation; “n.d.” indicates that the provider did not publicly disclose a cutoff date in a verifiable form.

Proprietary models were accessed via the respective vendor APIs. All proprietary models included in this study supported multimodal image input and an explicit native thinking or reasoning mode. Where exposed by the provider, this mode was enabled at the highest available setting. Exact model identifiers, including preview or date-stamped snapshot versions where applicable, are reported in Table 1 for transparency and version traceability. Directly comparable parameter counts were not used for proprietary models because these are generally not publicly disclosed in a standardized and verifiable form by providers.

Open-weight models were evaluated locally from publicly released weights using a standardized local inference pipeline. To capture a broad deployment-relevant range, open-weight models were stratified by publicly reported nominal parameter scale into models with fewer than 100B parameters and models with at least 100B parameters. For mixture-of-experts systems, these values refer to the total nominal parameter count reported by the model provider and do not imply that all parameters are active for any given token or inference step. This grouping was used as a practical distinction between smaller locally deployable systems and frontier-scale open-weight models rather than as a direct estimate of effective per-token compute. For local models that exposed an explicit native thinking or reasoning mode, this mode was enabled whenever available and configured to the highest supported setting. Open-weight base models without native thinking support were additionally evaluated under a two-stage chain-of-thought (CoT) prompting condition, as described below.

### Prompting strategies

All evaluations were conducted using zero-shot prompting in German to match the original examination language. Full prompt templates are provided in the Supplementary Material. No external tools, retrieval augmentation or task-specific prompt tuning were used. For multi-item case vignettes in M2, each item was presented together with its vignette text and the question texts and answer options of any preceding items within the same vignette; official solutions, model-selected answers from preceding items, and subsequent items within the same vignette were not provided. We defined a primary prompting condition applied across all models and an additional chain-of-thought condition for base models without native thinking support.

Under the primary constrained zero-shot condition, all models were instructed to return exclusively the single letter corresponding to the correct option (A–E), without additional explanation. This served as the main benchmarking condition throughout the study.

For base models without an explicit native thinking or reasoning mode, we additionally evaluated a chain-of-thought (CoT) condition by appending the German instruction *"Lass uns Schritt fuer Schritt vorgehen.”* ("Let’s think step by step.”). To standardize final answer extraction, this condition was implemented as a two-stage inference procedure: in the first stage, the model generated an unconstrained reasoning response; in the second, the same model was prompted to map this response to a single option letter (A–E). This condition was applied only to base models without native thinking support. Models with native thinking support were not prompted in this way, in order to avoid conflating built-in reasoning mechanisms with externally elicited reasoning formats.

### Inference setup

Benchmark evaluations were conducted between February and March 2026, using the model versions and configurations listed in Table 1. Unless otherwise specified, models were evaluated using provider-default inference settings or the corresponding default local pipeline settings. No additional decoding parameters were manually tuned or harmonized across models. The only systematic configuration change applied across models was the activation of native thinking or reasoning modes at the highest available setting where supported. These provider-specific reasoning or thinking settings should not be interpreted as equivalent inference-time compute budgets across systems. The resulting comparison is therefore a pragmatic evaluation of the evaluated model systems under standardized task prompts and commonly available default or maximum-supported reasoning configurations, rather than a compute-matched comparison of underlying model architectures.

For vision-capable models, images were provided in the native input format supported by the respective API or local inference pipeline. For OpenAI and Anthropic models, images were passed as base64-encoded inputs; for Google Gemini, as PIL image objects; and for locally deployed open-weight vision models, as the original image files. Images were provided in their original PNG or JPEG format. No auxiliary OCR, image captioning, or manual image-to-text conversion was introduced.

Text-only models did not receive image inputs under any condition. Accordingly, direct comparisons across the full model inventory were restricted to the shared text-only subset defined in Section Dataset and examination structure. Analyses involving all retained items, including image-present items, were restricted to vision-capable models.

Each model–item pair was evaluated once, without repeated sampling, ensembling, or majority voting. Requests were retried up to three times only in the event of transient API or transport errors or empty responses; retries were not triggered by answer content, formatting deviations, or incorrect predictions. Across the final benchmark runs, every model–item pair produced a non-empty generation that was not an API error, transport error, or content-policy refusal. No manual post-processing was performed beyond deterministic answer parsing.

### Answer extraction and scoring

Model outputs were normalized to uppercase and parsed using a deterministic regular-expression-based procedure to extract a single response option (A–E). Specifically, responses were scanned for the first occurrence of a valid option letter from A to E, while surrounding characters such as parentheses or other formatting symbols were ignored. This procedure therefore accommodated minor deviations from the constrained output format, including outputs such as *A)*, *(A)* or *A*. Parsing was applied identically across all models and prompting conditions, and no manual correction or adjudication of model outputs was performed.

As a robustness fallback, responses in which no valid option letter from A to E could be detected anywhere in the generation were scored as incorrect. In practice, this fallback did not apply to any response in the final benchmark runs. Accuracy was defined as the proportion of evaluated items for which the extracted model response matched the official IMPP answer key.

### Statistical analysis

The primary endpoint was accuracy, defined as the proportion of correctly answered items among all evaluated items. Accuracy was reported for the full benchmark, for the shared text-only subset, and for the image-present subset, as applicable. Because image inputs were available only for vision-capable models, direct comparisons across all models were restricted to the shared text-only subset. This subset comprised 3227 items in M1 and 3244 items in M2, with the stricter M2 definition reflecting the vignette-aware exclusion rule described in Section Dataset and examination structure. Analyses involving image-present items were restricted to vision-capable models and used all retained image-present items (M1 *n* = 520; M2 *n* = 432). In M2, the shared text-only subset and the image-present subset therefore do not exhaust the full benchmark, because a small number of no-image items were excluded from text-only analyses under the vignette-aware rule while remaining part of the full-benchmark evaluation.

Accuracy estimates were aggregated across examinations and weighted by item counts, corresponding to the total number of correct responses divided by the total number of evaluated items. We report two-sided 95% confidence intervals for accuracy using the Wilson score method. Mean student accuracy shown as a reference in figures was computed from the official item-level student correctness proportions over the corresponding item set. These official student performance statistics were derived from 119,878 examination sittings across the 24 included examination administrations.

To quantify modality-specific performance differences in vision-capable models, we computed two complementary metrics. The text–image gap was defined as the absolute difference in accuracy between text-only and image-present items, in percentage points. The error-rate ratio was defined as the ratio of the error rate on image-present items to the error rate on text-only items, (1 − acc_image_)/(1 − acc_text_).

To compare model difficulty with human difficulty at the item level, we used the official IMPP item difficulty index (*p*-value), defined as the proportion of students answering an item correctly. Human difficulty was operationalized as 1 − *p*. For this correlation analysis, model difficulty was defined as one minus the mean item-level correctness across the finalized text-only model pool used for the alignment analysis. Associations between human difficulty and model difficulty were assessed separately for M1 and M2 using Spearman correlation on eligible items from the shared text-only subset with valid official *p*-values, with 95% confidence intervals estimated by non-parametric bootstrap resampling of items (10,000 resamples).

To increase discriminatory power beyond near-ceiling overall accuracy, we additionally defined difficulty-enriched text-only subsets and analyzed them separately for M1 and M2. The human-hard-text subset comprised text-only items with an official student *p*-value of ≤ 0.30. The model-hard-text subset comprised text-only items on which at least two models from a top-tier comparison pool answered incorrectly. This pool included all proprietary frontier models and all open-weight models in the ≥100B size tier. These subsets were designed to capture complementary forms of difficulty: items that were exceptionally difficult for students and items that remained discriminative at the model frontier. Subset performance was summarized using accuracy and Wilson 95% confidence intervals. Overlap between difficulty-enriched subsets was assessed descriptively.

As an exploratory, non-reproductive probe of the human–model difficulty mismatch, we additionally performed an LLM-assisted qualitative characterization of the two off-diagonal cells of the cross-classification of human-hard-text and model-hard-text items (Suppl. Table S7). Within each cell, items were ranked continuously: items that were model-hard-text but not human-hard-text ("Set A”) by *p* × (1 − *a*), and items that were human-hard-text but not model-hard-text ("Set B”) by (1 − *p*) × *a*, where *p* is the official student correctness proportion and *a* is the mean accuracy of the top-tier comparison pool. The 10 highest-ranked items from each cell were retained. The two anonymized item sets were presented under neutral labels (Set A and Set B) to five of the evaluated models—GPT-5.4, Gemini 3.1 Pro, Claude Opus 4.6, GLM-5, and DeepSeek V3.2-Thinking—with no information about the underlying selection pattern and no predefined category scheme, so that categories had to emerge from the items themselves. Each model characterized the recurring properties and the competence probed by the questions in each set, and then contrasted the two sets. This procedure is exploratory and descriptive; it does not constitute a validated benchmark stratification or a formal multi-rater content annotation with inter-rater agreement. The prompt and the per-model summaries are reported in Suppl. Note 1.

As a secondary within-family analysis, we assessed temporal performance trends across successive OpenAI model generations under the same zero-shot evaluation protocol. For comparability across generations, reasoning-enabled OpenAI models in this secondary within-family analysis were evaluated using medium reasoning settings where applicable, rather than the highest available reasoning setting used in the main benchmark analyses. Unless otherwise stated, analyses were performed in Python (version 3.13.7) using standard libraries, including pandas and numpy.

## Supplementary information


Supplementary Information


## Data Availability

The datasets analyzed in this study were provided by IMPP and are not publicly available. They contain official examination materials, including item texts, answer options, answer keys, images, item identifiers, and psychometric metadata, which are subject to legal, contractual, copyright, and examination-security restrictions. Public release, including release in item-level or pseudonymized form, could enable reconstruction or redistribution of protected examination content. Access to the underlying examination materials is therefore controlled by the rights holder and cannot be granted by the authors. To support transparency, the manuscript and Supplementary Information report aggregate dataset accounting, exclusion counts, modality counts, model-level results, confidence intervals, and the definitions of all analysis subsets used in the study.

## Code availability

The analysis code is not publicly available because it is tightly coupled to restricted-access examination files, protected item identifiers, answer keys, image paths, and preprocessing steps specific to the IMPP source material. Releasing the code in executable form would not permit independent reproduction without access to the restricted datasets and could disclose details of the protected examination-material processing workflow. The computational procedures are described in the Methods and Supplementary Information, including item exclusion rules, text-only and image-present subset definitions, the M2 vignette-aware rule, prompt construction, answer extraction, scoring, confidence-interval calculation, contamination-probe analyses, and model grouping. Additional clarification of these procedures can be provided by the corresponding author upon reasonable request, subject to legal, contractual, copyright, and examination-security restrictions.
