# Supplementary material for: Evaluating foundation models on official German medical licensing examinations: Implications for high-stakes assessment and AI-assisted medical education
Source: NPJ Digit Med. 2026 Aug 31;9:670. doi: 10.1038/s41746-026-03082-7 (PMC13529676; doi:10.1038/s41746-026-03082-7)
Supplement: Supplementary file 1 [file 41746_2026_3082_MOESM1_ESM.pdf]

Supplementary Information

To document the exact evaluation procedure, prompts were dynamically constructed based on the structural properties of each item. All prompts were administered in German to match the official exam language. We detail the exact templates and the dynamic context-building for case vignettes below.

1. System Instruction

All models received the following system instruction to enforce constrained generation.

German (Original):

> *”Du bist ein medizinischer Assistent und sollst die folgende Multiple-Choice-Frage beantworten. Genau eine der fuenf Antwortoptionen ist richtig. Gib ausschliesslich den Loesungsbuchstaben (A, B, C, D oder E) aus und keine weiteren Informationen.”*

English Translation:

> *”You are a medical assistant and are supposed to answer the following multiple-choice question. Exactly one of the five answer options is correct. Output exclusively the solution letter (A, B, C, D, or E) and no further information.”*

2. Prompt Template: Standalone Items (M1 & M2)

For standalone items without an associated case vignette, the prompt was constructed using a single question block, followed by an answer prefix to cue the model's output.

Template:

```
[FRAGE]
<<<
{Question Text + Options A-E}
>>>

Antwort:
```

3. Prompt Template: Case Vignettes (M2)

For linked items based on a shared case vignette (Second State Examination, M2), models were provided with the overarching case description, the text and answer options of preceding questions within the same vignette, but not their official solutions or model-selected answers (to preserve clinical context), and the current question.

Template:

```
[FALLVIGNETTE]
<<<
{Vignette Text}
>>>
```

```
[BISHERIGE TEILFRAGEN]
<<<
Teilfrage 1:
{Text and answer options of preceding question 1 within the vignette}

Teilfrage 2:
{Text and answer options of preceding question 2 within the vignette}
>>>

[AKTUELLE FRAGE]
<<<
{Current Question Text + Options A-E}
>>>

Antwort:
```

Note: The `[BISHERIGE TEILFRAGEN]` block was dynamically generated. If an item was the first question of a vignette, this block was omitted.

4. Strategy B: Chain-of-Thought Condition (Base Models Only)

For base models evaluated under the chain-of-thought (CoT) condition, inference was implemented as a two-stage procedure. In the first stage, the standard constrained answer instruction was relaxed to allow free-form reasoning generation, and the prompt was augmented with a reasoning trigger.

Stage 1 system instruction:

> *"Du bist ein medizinischer Assistent und sollst die folgende Multiple-Choice-Frage beantworten. Genau eine der fuenf Antwortoptionen ist richtig."*

Stage 1 reasoning trigger:

> *"Lass uns Schritt fuer Schritt vorgehen."*

The resulting free-form reasoning output was then passed to a second-stage mapping step to obtain a standardized final solution letter. Native thinking models were not evaluated under this external CoT condition; instead, they were assessed using their native provider-specific reasoning or thinking configuration where available.

5. Final-answer mapping in the CoT condition

In the second stage of the CoT condition, the model's reasoning output from stage 1 was passed back to the same model for standardized final-answer mapping to a single option letter (A–E).

Stage 2 system instruction:

"Du bekommst eine medizinische Multiple-Choice-Frage und einen Antworttext. Deine Aufgabe ist es, aus dem Antworttext den gewaehlten Loesungsbuchstaben zu extrahieren. Gib ausschliesslich genau EINEN Grossbuchstaben aus {A, B, C, D, E} aus und keine weiteren Informationen. Falls kein Buchstabe explizit gewaehlt wurde, gib den Buchstaben aus, der der Antwort am ehesten entspricht."

Stage 2 prompt template:

```
[FRAGE]
<<<
{Question Context}
>>>

[ANTWORTTEXT]
<<<
{Reasoning Output}
>>>

Extrahiere den gewaehlten Loesungsbuchstaben aus dem Antworttext.
Antworte nur mit A, B, C, D oder E.
Antwort:
```

Table S1 Per-examination dataset accounting. Item-level accounting for all 24 examination administrations included in the benchmark.

Exam	Nominal items	Excluded items	Retained items	No-image items (raw)	Image-present
**M1**					
M1 2019 Spring	320	9	311	280	31
M1 2019 Fall	320	7	313	284	29
M1 2020 Spring	320	4	316	280	36
M1 2020 Fall	320	10	310	274	36
M1 2021 Spring	320	8	312	257	55
M1 2021 Fall	320	13	307	261	46
M1 2022 Spring	320	12	308	262	46
M1 2022 Fall	320	11	309	266	43
M1 2023 Spring	320	2	318	274	44
M1 2023 Fall	320	7	313	262	51
M1 2024 Spring	320	5	315	269	46
M1 2024 Fall	320	5	315	258	57
**Total M1**	**3840**	**93**	**3747**	**3227**	**520**
**M2**					
M2 2019 Spring	320	12	308	272	36
M2 2019 Fall	320	4	316	278	38
M2 2020 Spring	320	11	309	294	15
M2 2020 Fall	320	9	311	285	26
M2 2021 Spring	320	16	304	283	21
M2 2021 Fall	320	13	307	277	30
M2 2022 Spring	320	1	319	281	38
M2 2022 Fall	320	9	311	258	53
M2 2023 Spring	320	8	312	258	54
M2 2023 Fall	320	5	315	287	28
M2 2024 Spring	320	10	310	264	46
M2 2024 Fall	320	4	316	269	47
**Total M2**	**3840**	**102**	**3738**	**3306**	**432**
**Overall total**	**7680**	**195**	**7485**	**6533**	**952**

Note. Nominal items denotes the number of official item positions per examination before exclusion of invalidated items. No-image items (raw) denotes all retained items without an associated image. In M2, this count exceeds the shared text-only analysis subset because 62 additional no-image items were excluded after application of the vignette-aware rule to prevent within-vignette leakage.

Table S2 Full numerical model benchmark results for the main benchmark comparisons. Complete numerical model results for Figures 1 and 2. Panel A reports performance on the full benchmark, defined as all retained items, and is restricted to vision-capable models. Panel B reports performance on the shared text-only subset across all evaluated final model configurations. Values are reported as correct answers / total questions, followed by accuracy (%) and the corresponding 95% Wilson confidence interval.

Model	M1: k/n; accuracy [95% CI]	M2: k/n; accuracy [95% CI]
Panel A. Full benchmark (vision-capable models only)		
Proprietary models		
GPT-5.4	3690/3747; 98.48 [98.03–98.82]	3637/3738; 97.30 [96.73–97.77]
Gemini 3.1 Pro	3721/3747; 99.31 [98.99–99.53]	3677/3738; 98.37 [97.91–98.73]
Claude Opus 4.6	3632/3747; 96.93 [96.33–97.44]	3637/3738; 97.30 [96.73–97.77]
Open-weight models (≥100B parameters)		
Qwen3-VL	3539/3747; 94.45 [93.67–95.14]	3483/3738; 93.18 [92.32–93.94]
Mistral Large 3	3288/3747; 87.75 [86.66–88.76]	3414/3738; 91.33 [90.39–92.19]
Kimi K2.5	3624/3747; 96.72 [96.10–97.24]	3533/3738; 94.52 [93.74–95.20]
Open-weight models (<100B parameters)		
Gemma 3	2737/3747; 73.05 [71.60–74.44]	2904/3738; 77.69 [76.33–78.99]
Ministral 3	2870/3747; 76.59 [75.21–77.92]	2941/3738; 78.68 [77.34–79.96]
Panel B. Shared text-only subset (all models)		
Proprietary models		
GPT-5.4	3211/3227; 99.50 [99.20–99.69]	3180/3244; 98.03 [97.49–98.45]
Gemini 3.1 Pro	3215/3227; 99.63 [99.35–99.79]	3207/3244; 98.86 [98.43–99.17]
Claude Opus 4.6	3203/3227; 99.26 [98.90–99.50]	3184/3244; 98.15 [97.63–98.56]
Open-weight models (≥100B parameters)		
Qwen3-VL	3176/3227; 98.42 [97.93–98.80]	3073/3244; 94.73 [93.91–95.45]
MiniMax M2.5	3168/3227; 98.17 [97.65–98.58]	3082/3244; 95.01 [94.20–95.70]
Mistral Large 3	3048/3227; 94.45 [93.61–95.19]	3022/3244; 93.16 [92.24–93.98]
DeepSeek V3.2-Thinking	3186/3227; 98.73 [98.28–99.06]	3109/3244; 95.84 [95.10–96.47]
GLM-5	3198/3227; 99.10 [98.71–99.37]	3128/3244; 96.42 [95.73–97.01]
Kimi K2.5	3187/3227; 98.76 [98.32–99.09]	3106/3244; 95.75 [95.00–96.39]
gpt:oss 120B	3139/3227; 97.27 [96.65–97.78]	3041/3244; 93.74 [92.86–94.53]
Open-weight models (<100B parameters)		
Gemma 3	2540/3227; 78.71 [77.26–80.09]	2596/3244; 80.02 [78.61–81.36]
Ministral 3	2657/3227; 82.34 [80.98–83.61]	2616/3244; 80.64 [79.25–81.96]
gpt:oss 20B	3041/3227; 94.24 [93.38–94.99]	2907/3244; 89.61 [88.51–90.62]

Table S3 Modality-stratified performance by model in M1. Complete numerical results for the modality-stratified analysis in M1. Values are reported separately for text-only and image-present items as correct answers / total questions, followed by accuracy (%) and 95% Wilson confidence intervals. The absolute text–image gap is given in percentage points. The error-rate ratio is defined as $(1 - \mathrm{acc}_{\mathrm{image}})/(1 - \mathrm{acc}_{\mathrm{text}})$. Student reference values are computed from official IMPP item-level correctness proportions over the same item sets and are based on aggregated examination sittings; per-item k/n and Wilson confidence intervals are therefore not applicable.

Model	Text-only k/n; accuracy [95% CI]	Image-present k/n; accuracy [95% CI]	Gap (pp)	Error-rate ratio
GPT-5.4	3211/3227 99.50 [99.20–99.69]	479/520 92.12 [89.48–94.13]	7.39	15.90×
Gemini 3.1 Pro	3215/3227 99.63 [99.35–99.79]	506/520 97.31 [95.53–98.39]	2.32	7.24×
Claude Opus 4.6	3203/3227 99.26 [98.90–99.50]	429/520 82.50 [79.00–85.52]	16.76	23.53×
Kimi K2.5	3187/3227 98.76 [98.32–99.09]	437/520 84.04 [80.64–86.94]	14.72	12.88×
Qwen3-VL	3176/3227 98.42 [97.93–98.80]	363/520 69.81 [65.73–73.60]	28.61	19.10×
Mistral Large 3	3048/3227 94.45 [93.61–95.19]	240/520 46.15 [41.91–50.45]	48.30	9.71×
Ministral 3	2657/3227 82.34 [80.98–83.61]	213/520 40.96 [36.82–45.24]	41.37	3.34×
Gemma 3	2540/3227 78.71 [77.26–80.09]	197/520 37.88 [33.82–42.13]	40.83	2.92×
Students	– 71.21	– 64.27	6.94	1.24×

Table S4 Modality-stratified performance by model in M2. Complete numerical results for the modality-stratified analysis in M2. Values are reported separately for the vignette-aware text-only subset and for image-present items as correct answers / total questions, followed by accuracy (%) and 95% Wilson confidence intervals. The absolute text–image gap is given in percentage points. The error-rate ratio is defined as $(1 - \mathrm{acc}_{\mathrm{image}})/(1 - \mathrm{acc}_{\mathrm{text}})$. Student reference values are computed from official IMPP item-level correctness proportions over the same item sets and are based on aggregated examination sittings; per-item k/n and Wilson confidence intervals are therefore not applicable.

Model	Text-only k/n; accuracy [95% CI]	Image-present k/n; accuracy [95% CI]	Gap (pp)	Error-rate ratio
GPT-5.4	3180/3244 98.03 [97.49–98.45]	400/432 92.59 [89.73–94.70]	5.43	3.75×
Gemini 3.1 Pro	3207/3244 98.86 [98.43–99.17]	408/432 94.44 [91.87–96.24]	4.41	4.87×
Claude Opus 4.6	3184/3244 98.15 [97.63–98.56]	392/432 90.74 [87.64–93.13]	7.41	5.01×
Kimi K2.5	3106/3244 95.75 [95.00–96.39]	370/432 85.65 [82.03–88.64]	10.10	3.37×
Qwen3-VL	3073/3244 94.73 [93.91–95.45]	353/432 81.71 [77.79–85.07]	13.02	3.47×
Mistral Large 3	3022/3244 93.16 [92.24–93.98]	336/432 77.78 [73.62–81.44]	15.38	3.25×
Ministral 3	2616/3244 80.64 [79.25–81.96]	278/432 64.35 [59.73–68.72]	16.29	1.84×
Gemma 3	2596/3244 80.02 [78.61–81.36]	266/432 61.57 [56.90–66.04]	18.45	1.92×
Students	– 74.66	– 70.74	3.92	1.15×

Table S5 Performance of evaluated model configurations on the M1 difficulty-enriched text-only subsets. For each subset, values are reported as correct answers / total questions, followed by accuracy (%) and the corresponding 95% Wilson confidence interval. The human-hard-text subset was defined as text-only items with student $p \leq 0.30$. The model-hard-text subset was defined as text-only items on which at least two models from the top-tier comparison pool answered incorrectly.

Model	Human-hard-text	Model-hard-text
GPT-5.4	127/128 (99.2 [95.7–99.9])	79/93 (84.9 [76.3–90.8])
Gemini 3.1 Pro	126/128 (98.4 [94.5–99.6])	83/93 (89.2 [81.3–94.1])
Claude Opus 4.6	124/128 (96.9 [92.2–98.8])	75/93 (80.6 [71.5–87.4])
gpt:oss 120B	117/128 (91.4 [85.3–95.1])	38/93 (40.9 [31.4–51.0])
DeepSeek V3.2-Thinking	123/128 (96.1 [91.2–98.3])	55/93 (59.1 [49.0–68.6])
Kimi K2.5	122/128 (95.3 [90.2–97.8])	59/93 (63.4 [53.3–72.5])
MiniMax M2.5	119/128 (93.0 [87.2–96.3])	46/93 (49.5 [39.5–59.4])
GLM-5	124/128 (96.9 [92.2–98.8])	70/93 (75.3 [65.6–82.9])
Qwen3-VL	123/128 (96.1 [91.2–98.3])	52/93 (55.9 [45.8–65.6])
Mistral Large 3	114/128 (89.1 [82.5–93.4])	45/93 (48.4 [38.5–58.4])
gpt:oss 20B	106/128 (82.8 [75.3–88.4])	44/93 (47.3 [37.5–57.4])
Gemma 3	74/128 (57.8 [49.2–66.0])	33/93 (35.5 [26.5–45.6])
Ministral 3	80/128 (62.5 [53.9–70.4])	34/93 (36.6 [27.5–46.7])

Subset summary: human-hard-text $n = 128$, mean student $p = 0.221$; model-hard-text $n = 93$, mean student $p = 0.558$.

Table S6 Performance of evaluated model configurations on the M2 difficulty-enriched text-only subsets. For each subset, values are reported as correct answers / total questions, followed by accuracy (%) and the corresponding 95% Wilson confidence interval. The human-hard-text subset was defined as text-only items with student $p \leq 0.30$. The model-hard-text subset was defined as text-only items on which at least two models from the top-tier comparison pool answered incorrectly.

Model	Human-hard-text	Model-hard-text
GPT-5.4	189/197 (95.9 [92.2–97.9])	258/323 (79.9 [75.2–83.9])
Gemini 3.1 Pro	193/197 (98.0 [94.9–99.2])	288/323 (89.2 [85.3–92.1])
Claude Opus 4.6	189/197 (95.9 [92.2–97.9])	268/323 (83.0 [78.5–86.7])
gpt:oss 120B	162/197 (82.2 [76.3–86.9])	119/323 (36.8 [31.8–42.2])
DeepSeek V3.2-Thinking	179/197 (90.9 [86.0–94.1])	146/323 (45.2 [39.9–50.7])
Kimi K2.5	182/197 (92.4 [87.8–95.3])	194/323 (60.1 [54.6–65.2])
MiniMax M2.5	174/197 (88.3 [83.1–92.0])	132/323 (40.9 [35.7–46.3])
GLM-5	183/197 (92.9 [88.4–95.7])	168/323 (52.0 [46.6–57.4])
Qwen3-VL	166/197 (84.3 [78.5–88.6])	174/323 (53.9 [48.5–59.2])
Mistral Large 3	155/197 (78.7 [72.5–83.8])	174/323 (53.9 [48.5–59.2])
gpt:oss 20B	144/197 (73.1 [66.4–78.9])	113/323 (35.0 [30.0–40.3])
Gemma 3	102/197 (51.8 [44.9–58.6])	143/323 (44.3 [39.0–49.7])
Ministral 3	114/197 (57.9 [50.9–64.6])	138/323 (42.7 [37.5–48.2])

Subset summary: human-hard-text $n = 197$, mean student $p = 0.194$; model-hard-text $n = 323$, mean student $p = 0.633$.

Table S7 Cross-classification of human-hard-text and model-hard-text items. Text-only items were cross-classified according to the predefined subset definitions used in the main analyses. Human-hard-text was defined as items with student $p \leq 0.30$, and model-hard-text as items on which at least two models from the top-tier comparison pool answered incorrectly.

	**M1** ($n = 3{,}227$)			**M2** ($n = 3{,}244$)	
	**Model-hard**	**Model-hard^c**		**Model-hard**	**Model-hard^c**
**Human-hard**	12 (0.4%)	116 (3.6%)	**Human-hard**	40 (1.2%)	157 (4.8%)
**Human-hard^c**	81 (2.5%)	3018 (93.5%)	**Human-hard^c**	283 (8.7%)	2764 (85.2%)

Note. Superscript c denotes the complement of the corresponding predefined hard subset within the shared text-only subset. These complement labels should not be interpreted as separate easy-item categories. The overlap between the two predefined subset definitions comprised 12 items in M1 and 40 items in M2. Off-diagonal cells indicate disagreement between the two definitions.

Table S8 Empirical probes for direct benchmark contamination. For each evaluated model, the Spring-to-Fall 2024 accuracy difference on the shared text-only subset is reported as percentage-point difference followed by the 95% Newcombe score confidence interval. The exam-wise accuracy range is the difference between the maximum and minimum accuracy across the 12 administrations 2019–2024. Spearman ρ denotes the rank correlation between chronological examination index (1–12) and per-exam accuracy. Models are grouped by the temporal relationship between their reported pre-training cutoff and the 2024 examination administrations: cutoff-boundary-crossing models (cutoff between Spring and Fall 2024), post-cutoff models (cutoff after Fall 2024), and models without a publicly disclosed and verifiable cutoff ("n.d.").

**Model**	**Cutoff**	**M1 text-only**<br>Δ_{F-S} [95% CI]; range; ρ	**M2 text-only**<br>Δ_{F-S} [95% CI]; range; ρ
Cutoff-boundary-crossing (cutoff between Spring 2024 and Fall 2024)			
gpt:oss 20B	Jun 2024	−0.22 [−4.32, +3.79]; 2.21; +0.357	−1.03 [−6.14, +4.04]; 7.02; +0.105
gpt:oss 120B	Jun 2024	−0.14 [−3.57, +3.21]; 3.52; +0.119	−3.35 [−7.24, +0.14]; 7.12; +0.322
Gemma 3	Aug 2024	−0.86 [−7.84, +6.08]; 7.35; −0.070	−2.80 [−9.66, +4.01]; 8.23; −0.238
Post-cutoff (cutoff after Fall 2024)			
Gemini 3.1 Pro	Jan 2025	+0.74 [−0.82, +2.67]; 1.07; +0.080	−2.26 [−4.84, −0.80]; 2.26; +0.112
GPT-5.4	Aug 2025	+0.36 [−1.50, +2.31]; 1.09; −0.586	−3.76 [−6.78, −2.05]; 3.76; +0.028
Claude Opus 4.6	May 2025	−0.82 [−3.49, +1.54]; 1.94; −0.564	−2.62 [−5.65, −0.23]; 3.38; −0.294
No publicly disclosed cutoff (n.d.)			
Qwen3-VL	n.d.	−0.45 [−3.16, +2.08]; 2.35; −0.063	−1.82 [−6.21, +2.42]; 3.26; −0.203
MiniMax M2.5	n.d.	+2.20 [−0.28, +5.04]; 2.22; +0.333	−1.48 [−4.81, +1.61]; 5.38; +0.217
Mistral Large 3	n.d.	−1.04 [−5.62, +3.39]; 4.15; −0.378	−5.22 [−9.76, −1.09]; 5.84; −0.336
DeepSeek V3.2-Thinking	n.d.	+0.34 [−1.80, +2.53]; 2.33; +0.035	−4.11 [−8.14, −0.52]; 5.70; −0.280
GLM-5	n.d.	+0.34 [−1.80, +2.53]; 1.53; −0.273	−2.61 [−6.08, +0.44]; 5.35; +0.196
Kimi K2.5	n.d.	−0.44 [−2.95, +1.88]; 1.91; −0.329	−3.35 [−7.24, +0.14]; 4.99; −0.091
Ministral 3	n.d.	−0.36 [−6.97, +6.21]; 7.23; −0.018	−0.95 [−7.36, +5.45]; 11.11; +0.154

Note. Values are accuracy differences in percentage points (pp). Δ_{F-S} = Fall 2024 minus Spring 2024 accuracy on the shared text-only subset. Range = maximum minus minimum exam-wise accuracy across the 12 administrations 2019–2024. ρ = Spearman correlation between chronological examination index (1–12) and per-exam accuracy. Reported pre-training data cutoffs are taken from official provider documentation; "n.d." indicates that the provider did not publicly disclose a cutoff date in a verifiable form.

Table S9 Direct answering versus chain-of-thought prompting in base models for M1. Complete numerical results for the chain-of-thought (CoT) analysis in M1. Values are reported separately for direct constrained answering and chain-of-thought (CoT) prompting as correct answers / total questions, followed by accuracy (%) and 95% Wilson confidence intervals. Δ denotes the absolute accuracy gain under CoT prompting (CoT minus direct), in percentage points.

**Model**	**Direct**<br>**k/n; accuracy [95% CI]**	**CoT**<br>**k/n; accuracy [95% CI]**	**Δ**
Panel A. M1 overall			
Gemma 3	2737/3747<br>73.05 [71.60–74.44]	2933/3747<br>78.28 [76.93–79.57]	+5.23
Ministral 3	2870/3747<br>76.59 [75.21–77.92]	3228/3747<br>86.15 [85.01–87.22]	+9.55
Mistral Large 3	3288/3747<br>87.75 [86.66–88.76]	3441/3747<br>91.83 [90.91–92.67]	+4.08
Panel B. M1 text-only			
Gemma 3	2540/3227<br>78.71 [77.26–80.09]	2702/3227<br>83.73 [82.42–84.96]	+5.02
Ministral 3	2657/3227<br>82.34 [80.98–83.61]	2971/3227<br>92.07 [91.08–92.95]	+9.73
Mistral Large 3	3048/3227<br>94.45 [93.61–95.19]	3168/3227<br>98.17 [97.65–98.58]	+3.72
Panel C. M1 image-present			
Gemma 3	197/520<br>37.88 [33.82–42.13]	231/520<br>44.42 [40.21–48.72]	+6.54
Ministral 3	213/520<br>40.96 [36.82–45.24]	257/520<br>49.42 [45.15–53.71]	+8.46
Mistral Large 3	240/520<br>46.15 [41.91–50.45]	273/520<br>52.50 [48.21–56.76]	+6.35

Table S10 Direct answering versus chain-of-thought prompting in base models for M2. Complete numerical results for the chain-of-thought (CoT) analysis in M2. Values are reported separately for direct constrained answering and chain-of-thought (CoT) prompting as correct answers / total questions, followed by accuracy (%) and 95% Wilson confidence intervals. Δ denotes the absolute accuracy gain under CoT prompting (CoT minus direct), in percentage points. Panel B uses the same vignette-aware M2 text-only subset as the other M2 text-only analyses.

**Model**	**Direct**<br>**k/n; accuracy [95% CI]**	**CoT**<br>**k/n; accuracy [95% CI]**	**Δ**
Panel A. M2 overall			
Gemma 3	2904/3738<br>77.69 [76.33–78.99]	3011/3738<br>80.55 [79.25–81.79]	+2.86
Ministral 3	2941/3738<br>78.68 [77.34–79.96]	3173/3738<br>84.88 [83.70–86.00]	+6.21
Mistral Large 3	3414/3738<br>91.33 [90.39–92.19]	3492/3738<br>93.42 [92.58–94.17]	+2.09
Panel B. M2 text-only			
Gemma 3	2596/3244<br>80.02 [78.61–81.36]	2678/3244<br>82.55 [81.21–83.82]	+2.53
Ministral 3	2616/3244<br>80.64 [79.25–81.96]	2818/3244<br>86.87 [85.66–87.99]	+6.23
Mistral Large 3	3022/3244<br>93.16 [92.24–93.98]	3097/3244<br>95.47 [94.70–96.13]	+2.31
Panel C. M2 image-present			
Gemma 3	266/432<br>61.57 [56.90–66.04]	288/432<br>66.67 [62.09–70.95]	+5.09
Ministral 3	278/432<br>64.35 [59.73–68.72]	306/432<br>70.83 [66.38–74.92]	+6.48
Mistral Large 3	336/432<br>77.78 [73.62–81.44]	340/432<br>78.70 [74.60–82.30]	+0.93

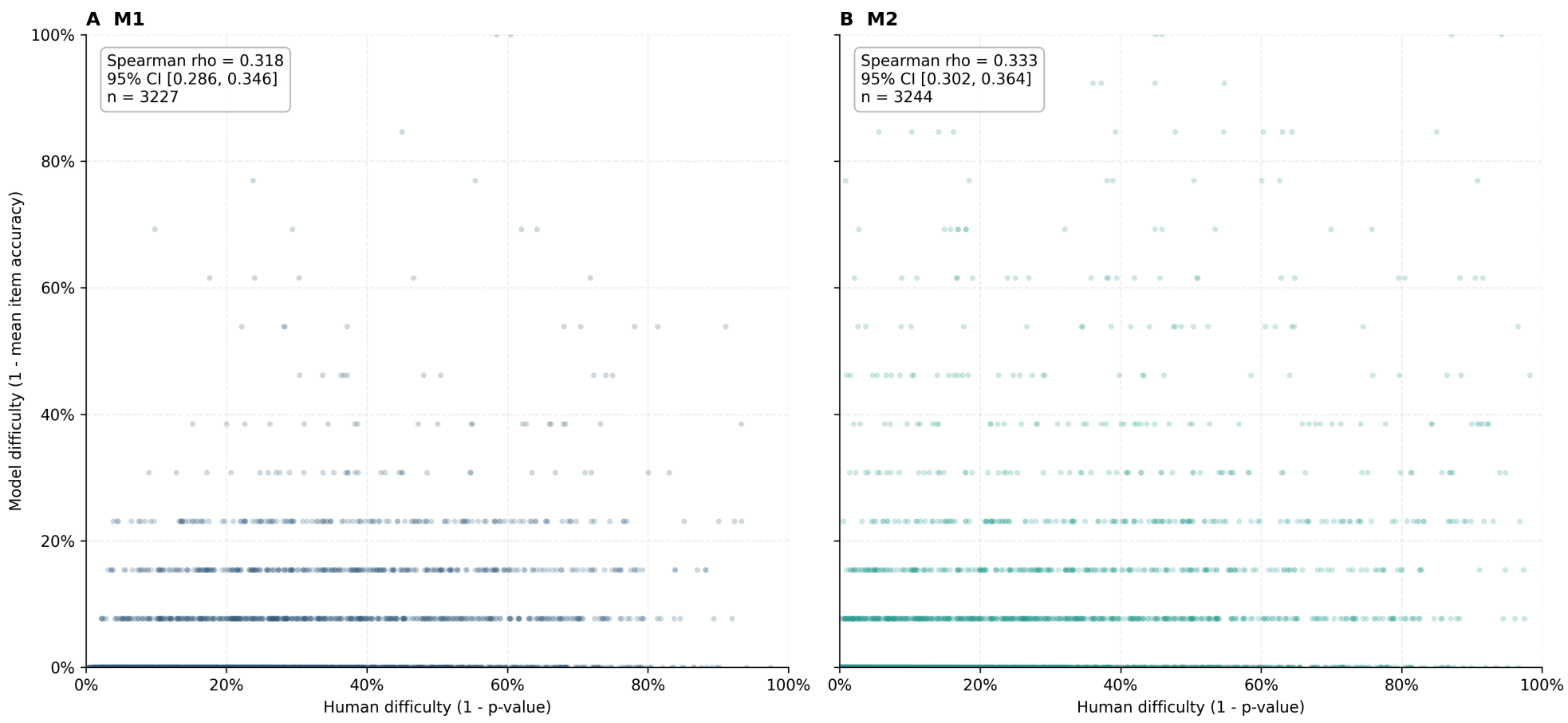

Fig. S1 Alignment between human difficulty and model difficulty. Item-level association between human difficulty and model difficulty on the eligible text-only subset for (A) M1 and (B) M2. Human difficulty is defined as $1 - p$, where p is the official student correctness proportion, and model difficulty is defined as one minus the mean item-level accuracy across the finalized text-only model pool used for the alignment analysis. Each point represents one item. Panels report Spearman ρ, 95% bootstrap confidence intervals, and the number of analyzed items included in the analysis.

A. M1 shared text-only subset across all final models

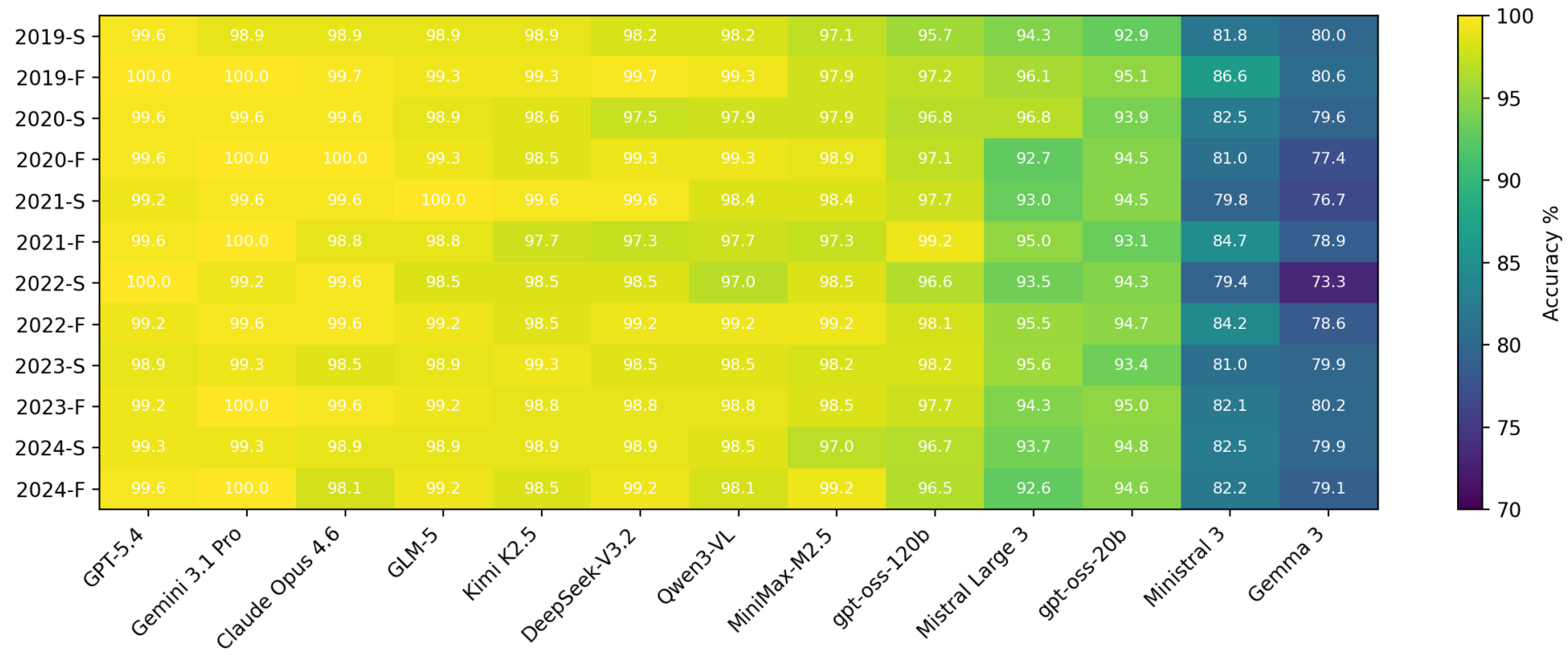

B. M1 full benchmark in vision-capable models

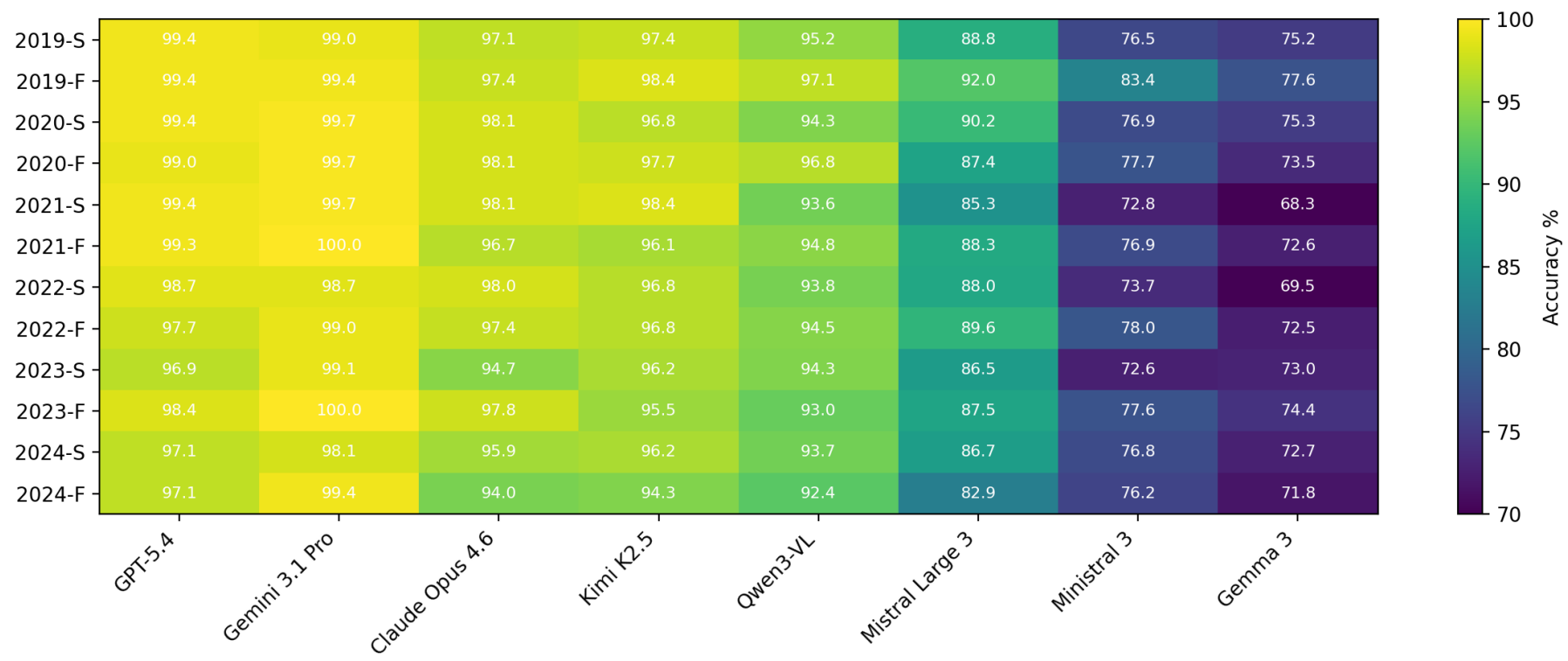

Fig. S2 Full exam-wise heatmaps for M1. Exam-wise accuracy across the 12 M1 examination administrations from 2019 to 2024. Columns correspond to the individual spring (S) and fall (F) examinations in chronological order. **A**, shared text-only subset across all final models, providing the fairest common basis for direct cross-model comparison. **B**, full benchmark, defined as all retained items, restricted to vision-capable models. Values are aggregated at the examination level and displayed as accuracy percentages.

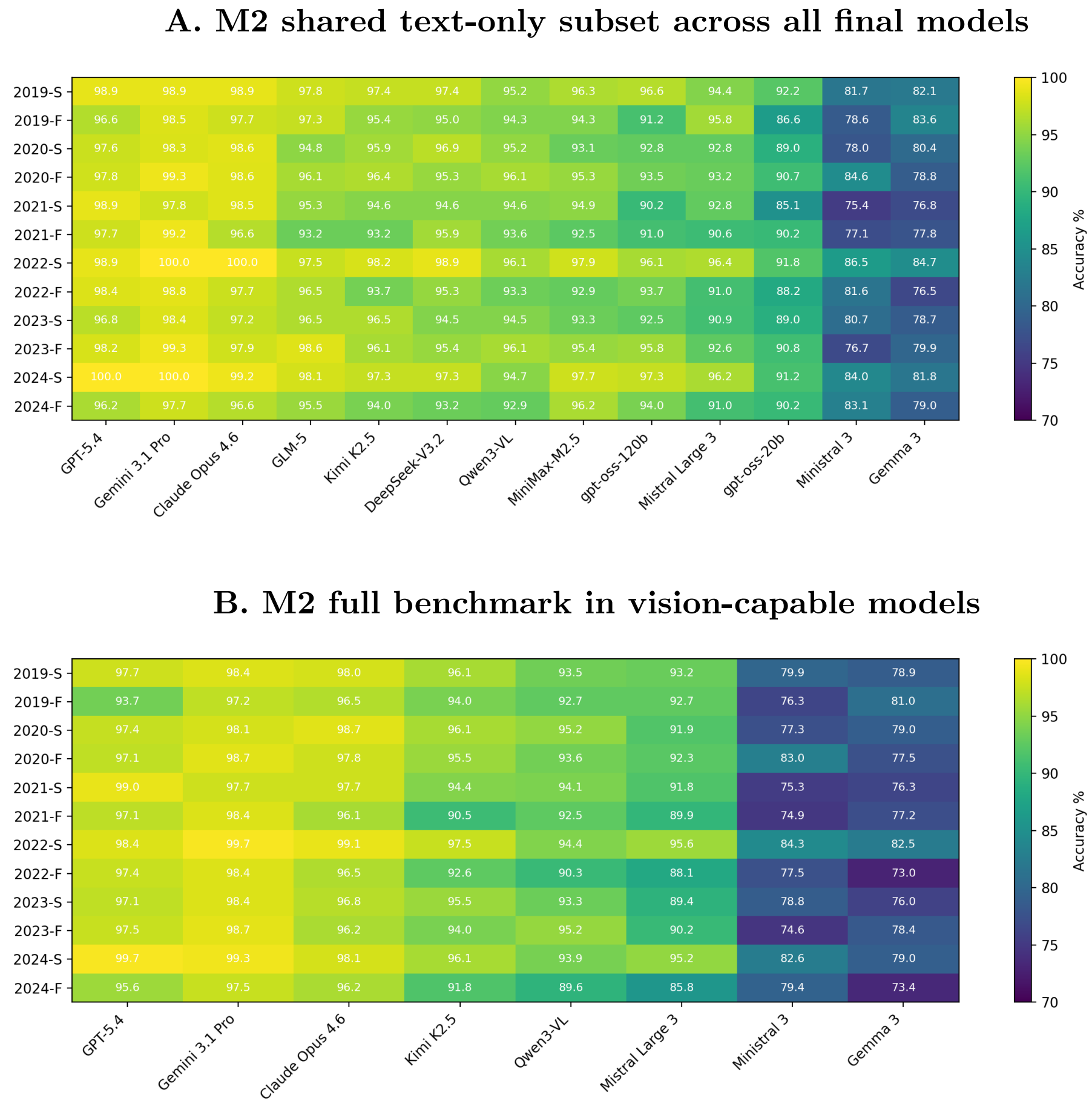

Fig. S3 Full exam-wise heatmaps for M2. Exam-wise accuracy across the 12 M2 examination administrations from 2019 to 2024. Columns correspond to the individual spring (S) and fall (F) examinations in chronological order. **A**, shared text-only subset across all final models, providing the fairest common basis for direct cross-model comparison. **B**, full benchmark, defined as all retained items, restricted to vision-capable models. Values are aggregated at the examination level and displayed as accuracy percentages.

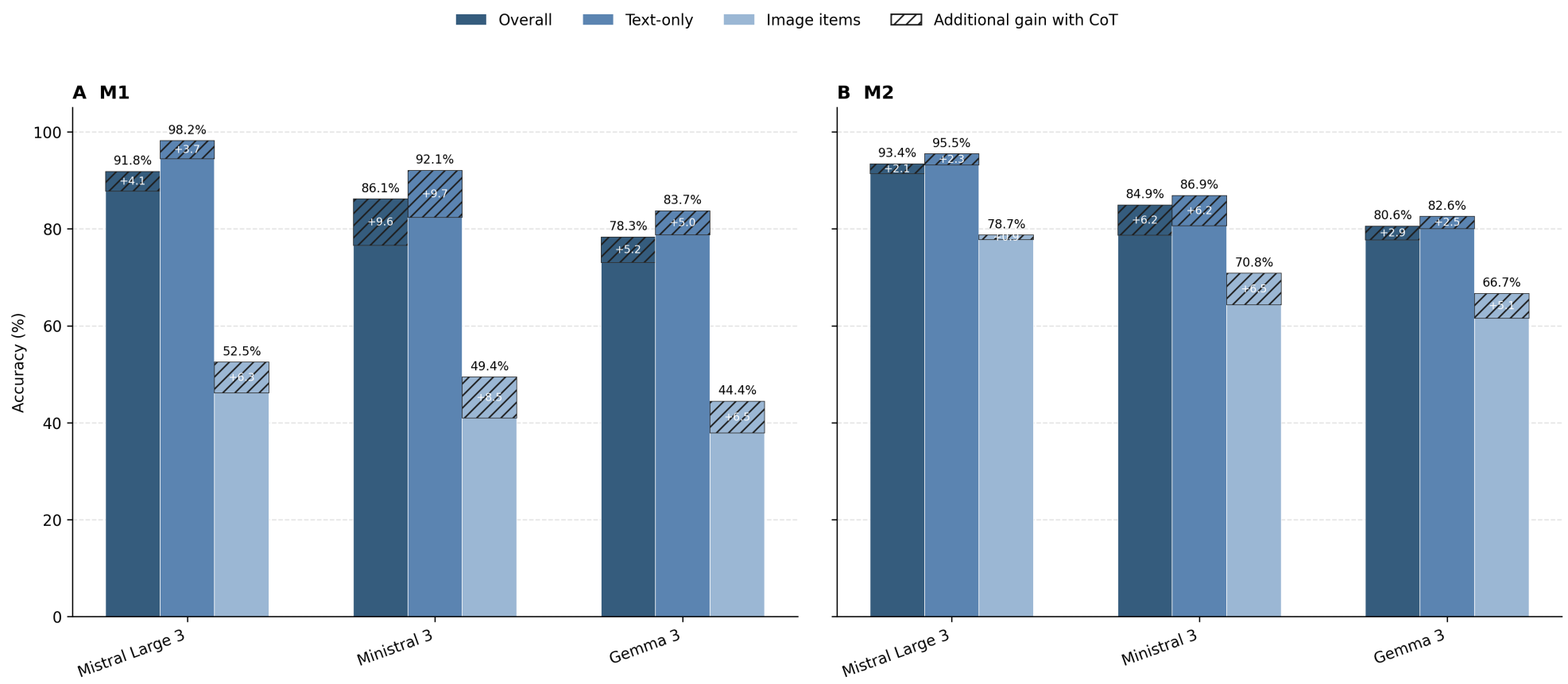

Fig. S4 Effect of chain-of-thought prompting in base models. Performance of base models with and without chain-of-thought (CoT) prompting on (A) M1 and (B) M2. Bars are shown separately for overall accuracy, accuracy on the shared text-only subset, and accuracy on image-present items. In each bar, the lower solid segment represents direct constrained answering, and the upper hatched segment represents the additional gain obtained with CoT prompting. The top of each stacked bar therefore corresponds to the final CoT accuracy. Models with native thinking support are excluded because they were evaluated using their built-in reasoning mode without external CoT prompting. Full numerical results are provided in Supplementary Tables S9 and S10.

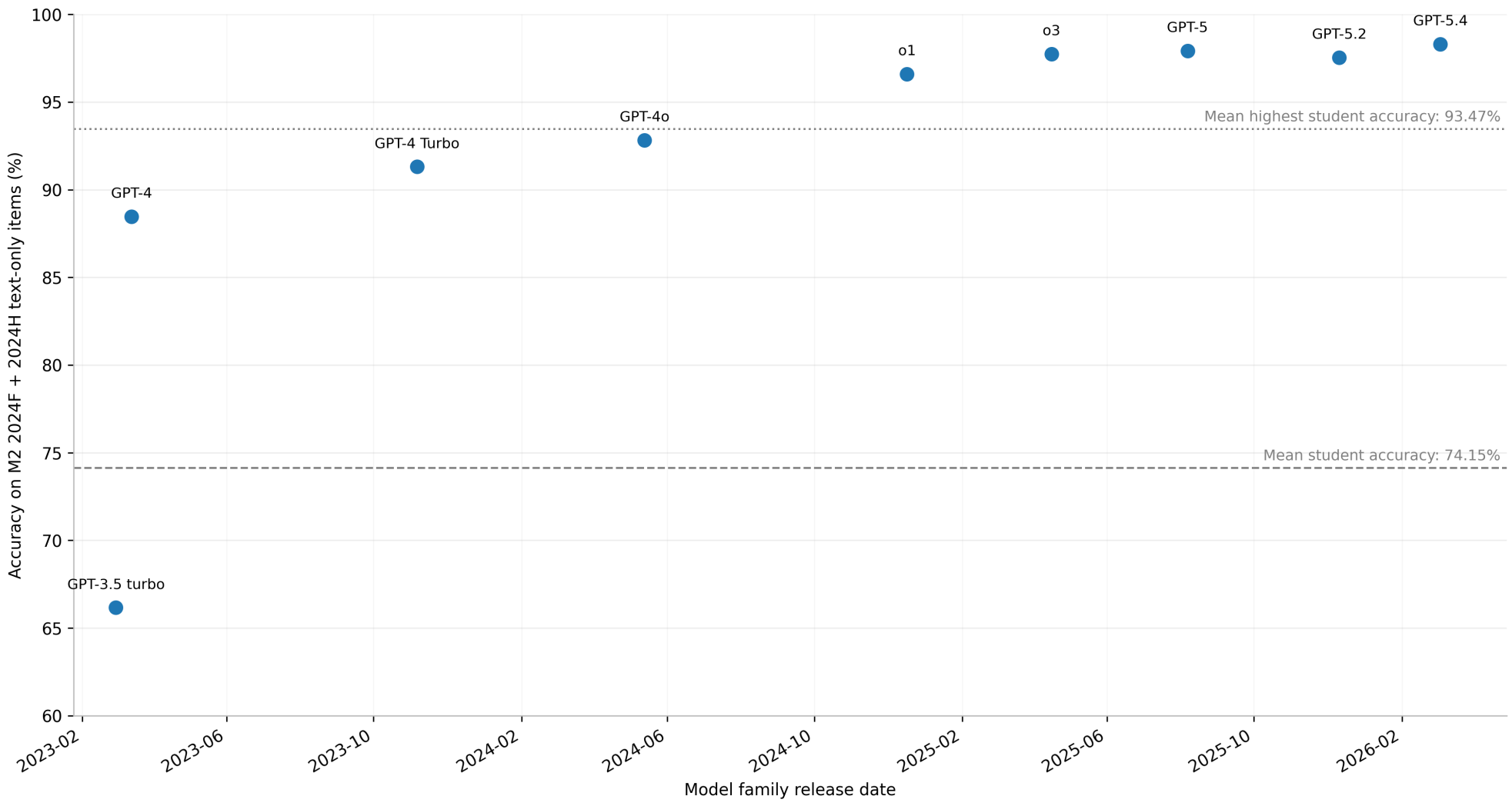

Fig. S5 Temporal performance trend within a single model family. Accuracy across successive OpenAI model generations on a fixed text-only subset comprising the two 2024 M2 examinations (spring and fall; $n = 529$ items). Each point represents one model generation, plotted against the release date of the corresponding model family. To improve comparability across model generations, reasoning-enabled models were evaluated using medium reasoning settings where applicable. Horizontal reference lines indicate the mean student accuracy across the two examinations and the mean of the highest observed student accuracies in the two examinations. This within-family comparison illustrates the marked performance increase from GPT-3.5 through later reasoning-enabled generations and the resulting compression toward near-ceiling benchmark performance.

Supplementary Note 1. Exploratory LLM-assisted qualitative characterization

As a limited exploratory probe of the human–model difficulty mismatch, we performed an LLM-assisted qualitative characterization of sampled discrepancy items, as described in the Methods section of the main manuscript. Set A comprised items classified as model-hard but not human-hard, whereas Set B comprised items classified as human-hard but not model-hard.

Judge models were shown the original protected text-only examination items in each set using only the neutral labels Set A and Set B. They were not informed which human–model discrepancy pattern each set represented. Because the examination items are protected, the item texts shown to the judge models are not reproduced here. The prompt text is shown below. For brevity, only the final "Central differences" section of each judge-model response is reproduced. The first sentence of each excerpt corresponds to the required one-sentence summary and is shown in bold for readability.

Prompt

M1 opening sentence: You are assisting a qualitative analysis of questions from the German medical state licensing examination (M1 — the first state examination, taken after approximately two years of medical study; single-best-answer items with five options, A–E).

M2 opening sentence: You are assisting a qualitative analysis of questions from the German medical state licensing examination (M2 — the second state examination, taken after approximately five years of medical study; single-best-answer items with five options, A–E).

Remaining prompt, identical for M1 and M2:
You will receive two sets of 10 text-only questions each (no figures).
The questions are in German; write your analysis in English.
Your task is to characterize the two sets qualitatively and independently — that is, to identify recurring properties of the questions — and then to compare them. The goal is not to solve individual questions. Derive your categories solely from the questions themselves; do not apply any predefined classification scheme.
Item protection: the original questions must not be published. Therefore, in none of your responses may you quote or paraphrase specific questions, answer options, diagnoses, case descriptions, numerical values, or any other details from which individual items could be reconstructed. Remain at an abstract, categorical level throughout, and give no individual examples.
Address the following points, in this order and under these headings:
Set A
Describe the recurring properties of the questions in Set A.
Set B
Describe the recurring properties of the questions in Set B.
Central differences
Begin with a single sentence capturing the clearest difference between the sets. Then, in approximately 100–150 words, describe how Set A and Set B differ most clearly. If the

sets cannot be clearly distinguished, or if the differences are weak or ambiguous, state that explicitly.

M1 responses

GPT-5.4

Set A is more mixed and terminology-oriented, with a marked psychosocial component, whereas Set B is more uniformly biomedical and mechanistic.

The clearest distinction is the type of knowledge being elicited. Set A repeatedly asks examinees to classify described situations, behaviours, diagnostic steps, or structures using the correct label. Even when biomedical content appears, the question format tends to be compact and identification-based. Set B, by contrast, more often embeds the target knowledge in a biological, experimental, physiological, or therapeutic context and asks for the mechanism, source, or causal explanation. Its questions are more concentrated in preclinical natural-science domains and less dependent on psychosocial terminology. The difference is clear rather than marginal: Set A feels broader and more concept-taxonomic, while Set B feels narrower in disciplinary range but deeper in biomedical mechanism.

Gemini 3.1 Pro

The clearest difference between the sets is that Set A primarily tests the classification of descriptive scenarios into abstract theoretical concepts and sequences, whereas Set B heavily emphasizes the direct recall of specific molecular, mechanistic, and physical facts.

Set A requires a more integrative cognitive approach, where examinees must interpret contextual vignettes, behavioral descriptions, or functional relationships to identify corresponding terminology, observational biases, or procedural orders. It leans heavily toward conceptual mapping, behavioral sciences, and applied methodology. In contrast, Set B is characterized by a high degree of granularity, focusing on the micro-level properties of the basic medical sciences. It demands the exact identification of specific substances, discrete modes of action, physical calculations, or biochemical properties based on precise functional descriptions. While Set A often asks examinees to interpret "what phenomenon is occurring" within a broader situation, Set B typically isolates a single variable and asks "what specific entity or mechanism" is responsible.

Claude Opus 4.6

Set A is predominantly classificatory — asking examinees to assign scenarios to the correct concept label — whereas Set B is predominantly mechanistic, requiring identification of causal processes, molecular targets, or quantitative reasoning.

More specifically, Set A leans heavily on medical psychology and social medicine, with items that present a vignette and ask "what is this called?" The cognitive task is recognizing which term from a taxonomy fits. Its biomedical items similarly tend toward single-fact recall. Set B, by contrast, is anchored in the natural sciences (biochemistry, physiology, physics, pharmacology) and asks "why does this happen?" or "how does this work?" The stems are often longer and richer in mechanistic or quantitative detail, and the distractors represent competing explanatory mechanisms rather than sibling labels. Where Set A tests

vocabulary within a classification system, Set B tests understanding of causal chains and functional relationships. The disciplinary balance also differs markedly: Set A overrepresents behavioral sciences, while Set B is almost entirely drawn from the biomedical and physical sciences.

GLM-5

The most distinct difference is that Set A focuses on defining and classifying psychosocial concepts and anatomical facts, whereas Set B prioritizes explaining scientific mechanisms and applying basic scientific principles.

In Set A, the cognitive demand is largely centered on the correct labeling of phenomena. A recurring theme is the identification of specific psychological terms for behavioral patterns or judgment errors, requiring the examinee to match a definition to a scenario. Even in the natural science questions, the focus remains on identifying structures or following defined sequences. In contrast, Set B requires a deeper understanding of functional relationships. The items in Set B ask "why" or "how" something occurs—whether it is the mechanism of a drug, the physical basis of a pressure change, or the biochemical rationale for an experimental substitution. Set A tests the recognition of established terms and facts, while Set B tests the understanding of dynamic processes and the application of scientific logic to solve problems.

DeepSeek V3.2-Thinking

The clearest difference between the sets is that Set A primarily tests the recall and application of defined concepts, structures, and sequences, whereas Set B tests the application of fundamental scientific principles to solve problems.

In approximately 100-150 words: Set A questions are largely concept- and structure-oriented, often centered on psychology, anatomy, and diagnostic logic, requiring the identification of correct terminology or the order of steps within a described scenario. In contrast, Set B questions are predominantly principle- and mechanism-oriented, drawing from biochemistry, physiology, physics, and pharmacology; they frequently present a scenario requiring the examinee to apply a specific scientific rule, calculation, or mechanistic insight. While both sets use clinical vignettes, Set A's vignettes typically set the stage for a conceptual classification, while Set B's vignettes often pose a concrete problem solvable through theoretical knowledge. The distinction in disciplinary focus and cognitive task (defining vs. applying principles) is clear and consistent across the items.

M2 responses

GPT-5.4

The clearest difference is that Set A mainly tests procedural judgment in context, whereas Set B mainly tests disease-specific recognition and standard biomedical knowledge.

The distinction is fairly clear, though not absolute. Set A repeatedly asks the examinee to act within systems, rules, workflows, and professional practice conventions: choosing the correct next measure, applying formal standards, or navigating institutional and procedural correctness. Its difficulty often lies in prioritization and relevance filtering within realistic, practice-oriented scenarios. Set B, by contrast, is more tightly organized around recognizable clinical patterns and canonical associations: matching presentations to diagnoses, tests, specimens, mechanisms, or standard treatment strategies. Its difficulty lies less

in navigating process and more in retrieving and discriminating among specialized medical facts. In that sense, Set A feels more operational and practice-regulatory, while Set B feels more specialty-biomedical and recognition-based.

Gemini 3.1 Pro

The clearest difference between the two sets is that Set A evaluates the administrative, legal, and systemic frameworks governing medical practice, whereas Set B strictly assesses pathophysiological reasoning and direct disease management.

Set A positions the physician within a broader healthcare system. Its items require an understanding of systemic protocols, legal obligations, ethical constraints, and administrative pathways, such as navigating insurance structures or adhering to standardized documentation and public health regulations. The clinical scenarios in Set A serve mostly as a backdrop for procedural or systemic decision-making. In stark contrast, Set B isolates the physician-patient-disease triad. The questions demand deep biomedical knowledge, challenging the examinee to interpret specific clinical signs, select exact diagnostic modalities, and identify optimal, disease-modifying treatments or pathological mechanisms. While Set A tests the rules, systems, and procedures of practicing medicine, Set B rigorously tests the biological science of diagnosing and treating illness.

Claude Opus 4.6

Set A emphasizes practical clinical management and procedural decision-making within detailed, complex scenarios, while Set B emphasizes biomedical knowledge recall and diagnostic pattern recognition within more concise presentations.

Set A's questions are built around lengthy vignettes laden with contextual detail — comorbidities, medications, social circumstances, and sometimes interpersonal conflict — requiring the examinee to navigate complexity and select an appropriate management action. Many items integrate non-biomedical domains such as medicolegal obligations, insurance systems, ethical principles, and documentation standards. The cognitive demand centers on judgment: filtering relevant from irrelevant information and applying procedural knowledge. Set B, by contrast, presents tighter clinical snapshots and asks examinees to identify diagnoses, select the correct diagnostic test, or recall specific pathophysiological facts. The reasoning is more convergent — recognizing a classic presentation or knowing which parameter matters — and the vignettes carry less extraneous detail. The two sets thus differ primarily in the type of reasoning elicited: applied clinical judgment versus focused biomedical recognition.

GLM-5

Set A focuses on the integration of legal, ethical, and administrative knowledge with clinical practice, whereas Set B prioritizes specialized diagnostic and therapeutic decision-making.

In Set A, the candidate is frequently tested as a professional actor within the healthcare system, requiring knowledge of documentation standards, reporting obligations, insurance structures, and ethical reasoning. The clinical context often serves as a vehicle to test procedural correctness or professional conduct. In contrast, Set B tests the candidate strictly

as a medical expert, requiring the recall of specialized pathological knowledge, the interpretation of specific diagnostic parameters, and the application of guideline-based therapies for defined diseases. While Set A encompasses the breadth of a generalist's responsibilities (including legal and social dimensions), Set B delves into the depth of specialist knowledge, focusing on the biological and technical aspects of patient care. Consequently, Set A questions often have a "best practice management" flavor, while Set B questions lean towards "identify the diagnosis or treatment protocol."

DeepSeek V3.2-Thinking

The clearest distinction is that Set A tests integrative clinical reasoning within complex, multifaceted scenarios, while Set B tests focused medical knowledge applied to more defined diagnostic or therapeutic problems.

Set A's items are characterized by elaborate vignettes simulating real-world clinical encounters where the examinee must navigate a web of patient-specific details, prior interventions, and sometimes ethical or administrative constraints to determine the best course of action. The challenge lies in synthesizing this information. In contrast, Set B's items are more streamlined, presenting a core set of findings—often a classic constellation of symptoms or a specific test indication—that point directly toward a single correct answer based on established medical facts. Set A evaluates the process of decision-making in context, whereas Set B evaluates the accurate identification of diagnoses, tests, or mechanisms. The difference lies in the cognitive task: synthesis and judgment versus recognition and recall.
